# Venomous Cargo: Diverse Toxin-Related Proteins Are Associated with Extracellular Vesicles in Parasitoid Wasp Venom

**DOI:** 10.3390/pathogens14030255

**Published:** 2025-03-05

**Authors:** Jennifer Chou, Michael Z. Li, Brian Wey, Mubasshir Mumtaz, Johnny R. Ramroop, Shaneen Singh, Shubha Govind

**Affiliations:** 1 Department of Biology, The City College of New York, New York, NY 10031, USA; 2 Department of Biology, Brooklyn College, Brooklyn, NY 11210, USA; 3 PhD Program in Biology, The Graduate Center, City University of New York, New York, NY 10016, USA; 4 PhD Program in Biochemistry, The Graduate Center, City University of New York, New York, NY 10016, USA

**Keywords:** annexin, extracellular vesicles, hemocyte, host, immune suppression, innate immunity, insect, parasitoid, toxin, venom, wasp, *Drosophila melanogaster*, *Leptopilina heterotoma*, *Leptopilina boulardi*

## Abstract

Unusual membrane-bound particles are present in the venom of the parasitoid wasps that parasitize *Drosophila melanogaster*. These venom particles harbor about 400 proteins and suppress the encapsulation of a wasp egg. Whereas the proteins in the particles of *Leptopilina boulardi* venom modify host hemocyte properties, those in *L. heterotoma* kill host hemocytes. The mechanisms underlying this differential effect are not well understood. The proteome of the *L. heterotoma* venom particles has been described before, but that of *L. boulardi* has not been similarly examined. Using sequence-based programs, we report the presence of conserved proteins in both proteomes with strong enrichment in the endomembrane and exosomal cell components. Extracellular vesicle markers are present in both proteomes, as are numerous toxins. Both proteomes also contain proteins lacking any annotation. Among these, we identified the proteins with structural similarity to the ADP-ribosyltransferase enzymes involved in bacterial virulence. We propose that invertebrate fluids like parasitoid venom contain functional extracellular vesicles that deliver toxins and virulence factors from a parasite to a host. Furthermore, the presence of such vesicles may not be uncommon in the venom of other animals. An experimental verification of the predicted toxin functions will clarify the cellular mechanisms underlying successful parasitism.

## 1. Introduction

Parasitoid wasps constitute a large group of highly successful insects with diverse and innovative strategies to ward off immune and other host defense mechanisms. These strategies enable the parasites to initiate their life cycle in their insect hosts, grow within them, and eventually kill them [1]. Fruit flies of the well-characterized genus Drosophila serve as hosts to over 60 parasitoid wasp species [2]. The flies of some *Drosophila* species defend themselves by encapsulating and melanizing wasp eggs. Wasp oviposition triggers these innate immune responses, which are carried out mainly by the blood cells called hemocytes [3,4].

Female parasitoid wasps of many lineages produce different types of particles in their venom or calyx fluid (e.g., [1,5,6,7]). These particles are introduced into the host during oviposition. The particles include domesticated viruses, endogenous viral elements, and virus-like particles (VLPs) [8,9]. The genes encoding most of their constituent proteins are present in the wasp genomes. In many cases, these particles can modify hemocyte properties, inhibit immune gene expression, and block melanization and capsule formation [6,10,11,12,13]. Venom particles from only a limited number of species have been characterized, considering the abundance and diversity of parasitoid wasps [1]

In this study, we compared the protein composition of venom particles from two figitid parasitic wasps that differ in their effects on *D. melanogaster* larval hemocytes. *Leptopilina boulardi* (*Lb*) are specialist parasites and fail to parasitize hosts beyond the melanogaster group of fruit flies. *L. heterotoma* (*Lh*) wasps are generalist parasites, successful on many *Drosophila* species [14]. The venom of both wasps contains particles, which are tentatively considered to be similar to extracellular vesicles (EVs) [15,16,17]. Previously called VLPs (also referred to as venosomes), the venom particles are large (>300 nm) and exhibit membranous projections [18,19,20]. Their proteins are expressed in the venom gland cells but their assembly appears to occur, at least in part, outside of the secretory cells, which express the venom proteins. Immune-electron microscopy studies reveal the presence of electron-dense particles outside the secretory cells, that do not resemble the morphology of the venom particles that are associated with host hemocytes [19,20,21]. Thus, the *Leptopilina* venom particles are unusual not only due to the presence of spiky projections but also because of their mode of biogenesis. There is no evidence that the venom particles from either species possess nucleic acid, a genome, or that they replicate in either the wasp or the host. The genes coding *Lb* and *Lh* venom particles are encoded in the wasp genome [16,17,22]. For these reasons, their biotic nature remains unclear.

We designed this study with the aim of uncovering the similarities and differences in venom particle proteomes. We expected to discover novel insights into the nature of this class of venom particles and simultaneously identify specific shared and unique virulence effectors. Venom particles from both species target two of the three hemocyte types present in *D. melanogaster* larvae (lamellocytes and plasmatocytes). While *Lb* venom particles distort lamellocyte morphology via a RhoGAP protein that is present in the *Lb* particles [23,24,25], the *Lh* particles destroy larval lamellocytes, plasmatocytes, and their progenitors, housed in the larval lymph gland. A virulence protein associated with the *Lh* venom particles (SSp40), not found in *Lb*, contributes to this process [26,27]. Plasmatocytes are phagocytic and make up almost all of the circulating hemocytes in unparasitized *Drosophila* larvae. Lamellocytes are not phagocytic and differentiate from hematopoietic progenitors in response to parasitization. Venom particles are endocytosed by plasmatocytes through the Rab5/Rab7 pathways, whereas they enter lamellocytes via a lipid raft-dependent mechanism [24,27]. Plasmatocytes and lamellocytes collaborate to encapsulate and melanize a wasp egg. *Leptopilina* wasps use their venom particles to foil encapsulation by promoting hemocyte modification and/or hemocyte elimination [3]. Thus, the venom particles from the two wasps differ in their mechanisms of immune suppression.

All prokaryotic and eukaryotic cells secrete diverse, membrane-bound forms of EVs. Animal cells produce two EV types that differ in their size and cargos, reflecting their distinct modes of biogenesis: the larger ectosomes (also known as the microvesicles) bud from the cell’s plasma membrane, while the smaller exosomes are derived from endosomal vesicles that fuse with the plasma membrane. The proteins required for their formation and release have been identified in invertebrate model systems [28]. Recent studies have also identified the protein markers associated with mammalian EVs [29,30,31], which is where the bulk of research work has been performed. Like mammalian EVs, a phospholipid bilayer surrounds *Lh* venom particles [16].

Our proteomic profiling of venom gland fractions enriched in *Lh* venom particles showed that they are similar to mammalian extracellular vesicles (EVs) [16,32]. We have used the *Lh* particle protein sequences to scan the transcripts expressed in the *Lb* venom gland transcriptome [16], but a direct comparison of venom particle proteins from the two species has not been made. We hypothesized that the particles from both wasp species should exhibit an overall similarity in their proteomic profile to reflect their shared biogenesis and other cellular properties; yet, the differences in their constituent proteins should provide clues to their differential effects on hemocytes. We addressed the following questions: (a) Do the venom particles contain the proteins that have been identified in mammalian ectosomes and exosomes? (b) Since particle biogenesis occurs in the parasites’ venom glands, do they contain the toxin-related proteins that are found in venomous organisms? (c) Might there be shared biochemical activities even in the proteins that are not conserved?

We identified many conserved domains in the proteomes of the *Lb* and *Lh* venom particles. Among these, we identified Annexin A1 homologs, a marker for mammalian microvesicles [29,31], in both proteomes. A large number and intriguing diversity of toxin-like proteins are present in both proteomes that have homologs in species ranging from bacteria to snakes. Finally, many unannotated proteins share structural similarities with bacterial ADP-ribosyltransferase toxins. Together, these findings suggest that the venom particles from both parasites possess properties of mammalian EVs, likely share biogenesis mechanisms, and serve to transfer a large variety of toxins and virulence factors into their hosts to potentially alter host physiology and ensure successful parasitism. Our results raise questions about the evolutionary origins of these particles and suggest that EVs may be common in invertebrate fluids such as parasitoid venom.

## 2. Methods and Materials

### 2.1. Venom Gland Morphology

Isogenized *L. heterotoma* strain 14 (*Lh14*) and *L. boulardi* strain 17 (*Lb17*) [14] were raised on the *y w* strain of *D. melanogaster*, which were reared on standard cornmeal, yeast, and agar media at 18 °C, as previously described [33]. Adult wasps were collected from parasitized hosts, 25 days after infection. Venom glands were carefully removed from female wasps by gently pulling out the ovipositors from their abdomen. Samples were fixed for 10 min at room temperature in 4% paraformaldehyde (Sigma P6148, Steinheim, Germany), prepared in phosphate-buffered saline (PBS, pH 7.4), washed in PBS, and stained with a mixture of rhodamine phalloidin (1 U/mL Life Technologies, Eugene, OR, USA, R415) and Hoechst 33258 pentahydrate (4 µg/mL; Invitrogen, Eugene, OR, USA, 1398) for 30 min. Samples were washed thrice in PBS and mounted in Vectashield for imaging using a laser scanning 800 confocal microscope (Carl Zeiss, Jena, Germany). Z-stacks of 6 µm thick scans through the organ were acquired first by detecting the top and bottom regions of each sample. The acquired Z-stack.czi image was opened in ZEN 3.5 (ZEN lite, blue edition) in the 3D mode. The DAPI channel was turned off, and a TIF image of the assembled venom gland was exported by selecting the ‘create image’ tab at a ‘display resolution’ of 1920 pixels × 1080 pixels.

### 2.2. Annotation

Venom particles from both wasps were isolated from the venom fluid as described in [16,34]. The proteomes of these purified venom particles were characterized in the liquid chromatography–mass spectrometry studies described before [16,34]. The 383 sequences in the *Lb* proteome were obtained from the *Lb* Gotheron strain [34], and were kindly provided by Dr. Julien Varaldi (University of Lyon). For the *Lh* proteome, a non-redundant list of 407 protein sequences was identified from our previous work on the *Lh14* and *LhNY* strains [16,17]. These *Lh* sequences are publicly available (Accession: GAJC00000000.1; [35]).

The protein sequences were uploaded to NCBI CDD (v3.20—59693 PSSMs or v3.21-62456) to query the conserved domains in batch mode with an E-value threshold of 0.01 (accessed in November 2023 and updated in January 2025). The results of the concise mode include three hit types: specific hits, a superfamily of the highest-ranking specific hits, and multi-domain models. The downloaded text files from CDD were imported into Excel to obtain a non-redundant list of the distinct conserved domains, from which the most abundant conserved domains and the unique conserved domains were identified in the two proteomes. The same *Lb* and *Lh* protein sequences were independently searched for in InterPro (v5.65-97.0) on November 2023 to validate the findings from the CDD searches. CDD domain assignments were also corroborated by our previous annotations using Blast2GO (v5.2; downloaded June 2018) [22].

### 2.3. Subcellular Localization Signals

The presence of a signal peptide and/or transmembrane domain in the two proteomes was examined utilizing the SignalP, TMHMM, and Phobius [36,37,38,39,40,41] programs, as described previously [17]. Assignments for each parameter were made only if a specific sequence met the criteria of both programs, i.e., SignalP and Phobius for a signal peptide, and TMHMM and Phobius for a transmembrane domain. For protein targeting within mitochondrial sub-compartments (outer membrane, intermembrane space, inner membrane, and the mitochondrial matrix), sequences were searched in DeepMito, a relatively new bioinformatic tool, trained on 424 mitochondrial proteins, whose sub-organelle localizations have been experimentally tested [42]. The sequences with less than 40 amino acid residues were removed for this search.

### 2.4. Enrichment Analysis

To determine if the *Lb* venom particles possess a proteomic profile similar to the profile described previously for the *Lh* venom particles [16,17], we performed an enrichment analysis for cellular components. The *Lb* and *Lh* proteins were included in the enrichment analyses only if a human ortholog was identified. The gene identifiers for the human orthologs of wasp proteins were obtained via BLAST searches on NCBI, which were compared against the UniProt database, and restricted to *Homo sapiens*. These orthologs (280 for *Lb* and 264 for *Lh*) were used as the input dataset to run FunRich’s enrichment analysis for cellular components. FunRich (v3.1.4) is a Functional Enrichment analysis tool which utilizes the Vesiclepedia database [43,44,45] (accessed July 2024). The Vesiclepedia database contains manually curated data of EVs and extracellular particles from a variety of species. The percentage of genes enriched in a specific cell compartment (e.g., plasma membrane, nucleus, etc.) was calculated by dividing the number of *Lb*/*Lh* orthologs identified by FunRich to be expressed in the cell compartment by the total number of orthologous *Lb*/*Lh* genes within the FunRich/Vesiclepedia database (270 for *Lb* and 254 for *Lh*). Fold enrichment for cellular components is the percentage of genes for a specific cellular component category divided by the corresponding percentage in the background database. Graphs illustrating the enrichment results of these *Lb* and *Lh* orthologs were drawn using FunRich and edited in Microsoft Excel. A simultaneous analysis of both datasets was performed to ensure that both proteomes were analyzed with the same available version of the database.

### 2.5. Multiple Sequence Alignment

Protein BLAST searches for the human homologs of wasp Annexins (g6263.t1 and GAJC01012766.1_49) were performed in the default settings using the NCBI’s BLASTP tool (accessed January 2025), which were compared against the nr protein sequence database, restricted to *Homo sapiens*. The human sequences were aligned with the wasp sequences using the EMBL-EBI’s T-Coffee program [46] and visualized using ESPript [47].

### 2.6. Venom Toxins

Manually curated toxin and venom proteins (7828 proteins), as part of the animal toxin annotation project [48,49], were downloaded from UniProt in August 2024. Non-animal toxin and venom proteins were downloaded from UniProt after modifying the search parameters as follows: (Taxonomy[OC] 33208, AND tissue specificity “toxin”, evidence “Any”, OR keyword[KW] KW-0800, AND reviewed “yes”). This returned 7787 proteins. A non-redundant list of 8724 proteins was made. Protein BLAST was performed using these toxin proteins as the subject and the venom particle proteins as the query using the default parameters (with an expected threshold of 0.05; accessed in September 2024). Results were considered high confidence if the E-value of the protein BLAST result was less than 1 × 10^−5^.

To predict the functions of venom particle proteins in the context of known venom or toxin functions, we compared the CDD identifiers in the wasp sequences and in the non-wasp toxin sequences identified in our BLAST search. For this, the toxin sequences were submitted to the NCBI Batch Web CD-Search Tool in December 2024 using the default parameters (automatic search mode; CDD—62456 PSSMs database; E-value threshold of 0.01; selected for composition-corrected scoring).

### 2.7. Bacterial ARTs

The *Lb* and *Lh* proteins that failed annotation were queried with pLM-BLAST (version ECOD90_20231201 [50]; accessed in February 2024), using the local alignment mode and an alignment score cut-off of 0.3. These results identified 44 sequences, 22 from each species, that carried the ART-related annotation, and these sequences were analyzed further. As expected, multiple sequence alignment efforts failed to identify significant sequence similarities in the 44 sequences with ART annotation. Hence, their corresponding predicted protein sequences from genomic assemblies (with XP identifiers) were identified as follows: Annotated genome assemblies for the *Lb* strain G486 and *Lh14* (accession numbers JADEYJ010000000.1 and JABAIE010000000.1, respectively, [51]) were downloaded in June 2024. The 44 *Lb* and *Lh* putative ARTs were queried to identify their genomic counterparts using NCBI TBLASTN (BLAST+ (v2.7.1)) [52,53,54]. The cut-off criteria for positive results were query coverage > 50%, %ID > 40%, and an E-value of 1 × 10^−7^.

The AlphaFold 3 web server [55] was then used to predict the 3D structures of all 44 primary sequences from the wasp proteomes and the 44 XP sequences, along with their confidence scores. Unlike the sequences from the proteomes, however, the XP sequences could be aligned with CLUSTAL Omega, and their AlphaFold structures showed higher confidence scores relative to the primary sequences from the proteomes. Hence, the XP sequences were selected for further examination.

To verify the presence of the ART tertiary fold, the AlphaFold structures were compared with the existing protein structures in the DALI server [56]. These DALI search results suggested that 36 (19 *Lb* and 17 *Lh*) out of the 44 sequences found in the pLM-BLAST searches were likely to contain the ART fold, while the remaining 8 (3 *Lb* and 5 *Lh*) sequences did not contain this fold. The 36 XP protein sequences were analyzed further for docking simulations with NAD^+^ as the ligand. This step was performed on the full-length AlphaFold-derived structures, using the AlphaFold 3 web server [55].

Only 4 of the 36 XP sequences met the following criteria: (a) high confidence scoring models (i.e., a predicted template modeling (pTM) score of 0.5 or better); (b) a single β-sandwich domain; and (c) if NAD^+^ docked specifically in the β sandwich with a score of 0.8 or better (Table S4). The β-sandwich domain was identified in the AlphaFold structures using PyMol version 3.0.2 [57], which was corroborated with the Stride secondary structure analysis webserver [58] accessed in November 2024. The results from the remaining sequences will be described elsewhere.

To identify the consensus signatures (R-S-E or H-Y-D) of the putative ARTs and locate the key NAD^+^-interacting amino acid residues from the docking scenarios in the full-length sequences, we extracted the single β-sandwich domain sequences from the AlphaFold structures and mapped the NAD^+^-interacting residues using PDBSum [59]. The extracted sequences from all four putative ARTs were aligned using Clustal Omega v1.2.4 [60]. The key amino acid residues identified using PDBSum [59] from the NAD^+^-docked complexes were visualized using ESPript v3.0 [47] on the Clustal Omega-generated multiple sequence alignment (November 2024). Structural similarity with a known R-S-E type ART, *Bacillus cereus* C3 exoenzyme (PDB ID: 4XSH; [61]), was assessed by aligning the structures in PyMol using its in-built structural alignment algorithm. C3 was chosen as a reference ART since it also possesses the R-S-E type of ART motif and is similar in size and in its secondary structure elements to the four putative ARTs being analyzed [62,63].

## 3. Results and Discussion

### 3.1. Venom Glands

**Figure 1:**
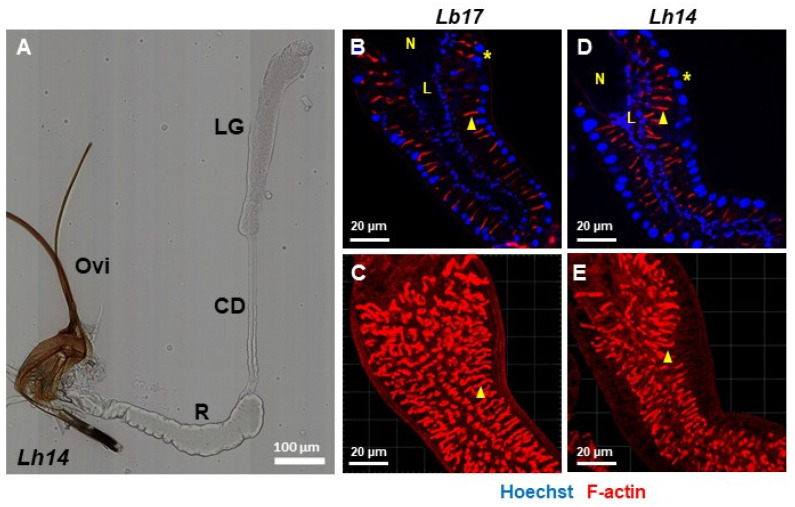
Venom gland morphology. (**A**) The entire venom gland complex is composed of the long gland (LG, anterior-most); the connecting duct (CD); the reservoir (R); and the ovipositor (Ovi). The ovipositor enters the host larva during egg laying, and the venom contents are deposited into the host at the same time. (**B**–**E**) The long glands from *Lb17* (**B**,**C**) and *Lh14* wasps (**D**,**E**), stained with Hoechst 33258 and rhodamine phalloidin. For all samples, the anterior end of the long gland is placed in the top left. N = nose; L = long gland lumen; * = secretory cells; triangle = canals. (**C**,**E**) Only the red channel is shown to highlight the F-actin-rich canals. Select Z-stack images were assembled from either 9 (for *Lb*), or 10 (for *Lh*) optical sections to visualize the 3D views of canal organization.

The entire venom gland complex from *Leptopilina* wasps is composed of a long gland–reservoir complex. The long gland (LG) continues into the narrow connecting duct (CD), which joins the reservoir (R), the latter of which is connected to the ovipositor (Ovi, Figure 1A; also see [15,19,64]). The female wasp inserts her ovipositor into the host larva for egg laying, and at the same time, deposits the venom contents inside the host’s body cavity. The long glands of both species are long and tubular and have a row of secretory cells (asterisks, Figure 1B,D) that are arranged peripherally, around a central lumen (L, Figure 1B,D). These cells have been shown to synthesize and secrete venom components.

Individual secretory cells are connected to the lumen by actin-rich canals (Figure 1B–E). Previous imaging studies have shown that the strong phalloidin signal in the long gland arises from the rough, actin-rich microvillar regions of the canal, which, in cross-sections, appear to contain the precursors of the venom particles [15,19,65]. Thus, the products of the secretory cells are first secreted into the lumen of the actin-rich canals before they join the common long gland lumen [19,65]. This general interpretation of particle biogenesis and identification of their target host cells is supported by the antibody staining studies in which at least one particle protein from each wasp has been shown to localize in the long gland secretory cells, and in host hemocytes [15,20,27,65]. This side-by-side comparison of the long glands from both wasps reveals a striking similarity in their overall organization despite the differences in their infection strategies. The particle biogenesis steps, and particle morphologies have been described in detail in previous studies [19,20].

### 3.2. Lb and Lh Venom Particles Share Proteomic Profiles

#### 3.2.1. General Findings

To compare the proteomic profiles of particles from *Lb* and *Lh* venom, we performed CDD and UniProt searches of their proteomes. We present our findings and interpretations in the context of NCBI’s conserved domains, with data presented in a concise format (Table S1) as this format presents the results from the best domain model with the lowest E-value for each region. The CDD hit results revealed that out of the 383 *Lb* particle proteins, 309 (80.68%) proteins have one or more conserved domains with an E-value of ≤0.01. The remaining 74 proteins did not have an identifiable conserved domain. Similarly, of 407 *Lh* proteins, 299 (73.46%) proteins were predicted to contain one or more conserved domains. The remaining 108 proteins were not assigned a conserved domain. When the proteins lacking a conserved domain with an E-value threshold of 0.01 were queried in UniProt, all of them remained without identifiable domains. The *Lb* and *Lh* proteomes share 110 protein domains.

A vast majority of the proteins in both proteomes contain just one conserved domain. Of the 309 *Lb* proteins, 247 (79.94%) have one CDD hit; the rest contain 2–8 domains. (Only one protein contains 8 domains; the rest have 2–5 conserved domains.) The proteins in the *Lh* proteome have a similar profile: 268 out of the 299 proteins (89.63%) have only one CDD hit, while the remaining proteins have 2–6 domains. Thus, even though the particle purification and peptide analysis for the two wasps were performed in different labs, there is a remarkable overall similarity in the complexity of the two proteomes and the proportions of proteins with and without conserved domains.

The searches for a signal peptide (SP) and transmembrane domain (TD) for each protein predicted 11/383 (2.9%) *Lb* and 12/407 (3%) *Lh* proteins to have both these protein signals. Thus, only a small number of proteins represent candidates for mediating interactions with the host’s (hemocyte) cell membrane.

Among the most abundant conserved domains in both proteomes are the RhoGAP superfamily proteins (cl02570; Table S1). These proteins regulate the activity of Rho family GTPases by accelerating the hydrolysis of GTP to GDP, which is crucial for regulating the cytoskeleton, cell migration, and cell adhesion. A RhoGAP from *L. boulardi* (LbGAP) inhibits wasp encapsulation [66], but the functions of the other family members are not known. The fibronectin-like FN3 domain (cd00063) previously described in *Lh* particles [16] is also represented in the *Lb* proteome (Table S1).

#### 3.2.2. Enrichment Analysis of Venom Particle Proteins and Localization Signals

A functional enrichment analysis involving the cellular components of the human orthologs of the wasp proteins found in Vesiclepedia revealed a similar trend: in both cases, statistically significant enrichment was observed for exosomes, the ER–Golgi intermediate compartment, mitochondria, ribosomes, and nucleosomes (*p* < 0.01), but not for the plasma membrane, nucleus, and cytoplasm (*p* > 0.01). Even though the percentage of genes in the endoplasmic reticulum–Golgi intermediate compartment is lower than in the other categories, the fold-enrichment for this category is high (4.07% of genes; 19.8-fold enrichment for *Lb*, and 2.36% of genes; 11.5-fold enrichment for *Lh*).

The proteins with these superfamily domains are expected to be part of the cell’s endomembrane system: Malectin (pfam11721); TRAP_alpha (pfam03896); ER_PDI_fam superfamily (cl36828); SNARE (cd15866); Ribophorin_I (pfam04597); and longin-like superfamily (cd14824).

**Figure 2:**
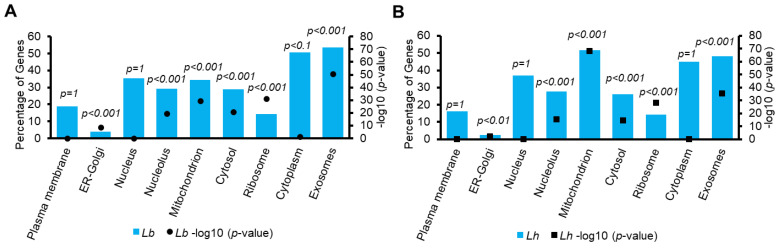
Characterization of *Lb* and *Lh* EV proteins. (**A**,**B**) Enrichment analysis of *Lb* (**A**) and *Lh* (**B**) EV proteins. The specific cellular compartments found in Vesiclepedia are shown on the X-axis. The primary Y-axis indicates the percentage of genes, calculated by FunRich, as the number of genes within the provided dataset (for *Lb* or *Lh*) that are associated with the listed cellular compartment (i.e., plasma membrane, nucleus, etc.) divided by the total number of genes within the provided dataset found within the FunRich/Vesiclepedia database (see Methods). The secondary Y-axis shows the −log10 (*p*-value). In both species, there is significant enrichment in the exosomes, lysosomes, mitochondria, ER–Golgi compartment, and ribosomes (*p* < 0.01). The Bonferroni correction was used by the FunRich program to calculate the *p*-values.

The percentage of genes in the exosomal category is 53.70%, with 3.8-fold enrichment, while 48.03% of the *Lh* genes in the same category showed 3.4-fold enrichment. Thus, consistent with our previous observations [16,17], the *Lb* venom particle proteome is also broadly similar to that of *Lh* venom particles in terms of the kinds of proteins included; the *Lb* proteome shows a slightly higher enrichment value for exosomes than the *Lh* proteome (Figure 2A,B).

The venom particles under consideration here are not likely to be exosomal in nature due to their large size and spiked morphology (exosomes are 30–150 nm wide and do not possess spikes, while wasp venom particles are ~300 nm). Given that extracellular vesicles from insects are not as well characterized as human EVs, we searched for the proteins that serve as markers of these categories in human samples.

A recent study by Jeppesen et al. utilized density gradient fractionation and immunoaffinity capture to reevaluate the composition of human exosomes and distinguish the exosomal proteome from that of microvesicles, as well as from nonvesicle compartments [29]. Another study examined the differences between exosomes and microvesicles found in the published literature including the Jeppesen study and identified potential markers [31]. Both studies verified that the classical mammalian tetraspanin markers for exosomes are CD63, CD81, and CD9, and further identified annexin A1 to be a specific marker for microvesicles. When searching for proteins with tetraspanin domains, we found that the *Lb* protein, g14457.t1, contains the tetraspanin protein domain, cd03127, while none of the *Lh* proteins show the presence of this domain.

Strikingly, one *Lb* protein (g6263.t1) and one *Lh* protein (GAJC01012766.1_49) carry four copies of the annexin domain (cl02574, pfam00191) and exhibit 59.9% sequence identity over their length (100% query coverage). The NCBI protein BLAST analysis of the *Lb* sequence revealed that g6263.t1 and human Annexin A1 (NP_000691.1) show 44.86% identity (at 92% query coverage and 6 × 10^−80^ E-value). Similarly, the *Lh* GAJC01012766.1_49 and human Annexin A1 show 46.10% identity (with 95% query coverage, and an E-value of 3 × 10^−80^) (Figure S1). The human Annexin 1 (ANXA1) binds to specific phospholipids in a Ca^2+^-dependent manner. It regulates actin dynamics and is involved in endosomal cargo sorting. Present inside and outside of cells, the ANXA1-mediated tethering of EVs encourages EV aggregation and promotes ectopic calcification and disease pathology [67].

One *Lh* protein (GAJC01011407.1_9) has the SPFH_like superfamily domain (cl19107), with the cd03401 prohibitin domain. Prohibitin is an exosomal marker in some systems [68]. Overall, these results support the interpretation that, like the *Lh* venom particles [16,17], the *Lb* venom particles also possess an extracellular vesicle-like character.

Our enrichment analysis results revealed the existence of numerous proteins in the mitochondrial and ribosomal categories. Accordingly, we found annotations for many metabolic enzymes in both proteomes. Mitochondrial targeting signals were found in 67 *Lb* proteins as follows: 40.30% to the matrix, 26.87% to the inner membrane, 17.91% to the intermembrane space, and 14.93% to the outer membrane. The corresponding proportions for putative mitochondrial localization signals of the 102 *Lh* proteins are as follows: 33.33% to the matrix, 40.20% to the inner membrane, 17.65% to the intermembrane space, and 8.82% to the outer membrane. Of the ~110 protein domains in both proteomes, there is also an abundance of ribosomal protein domains with at least 12 ribosomal annotations in each proteome (Table S1).

The cytoskeleton-associated protein domains, e.g., tubulin alpha chain (PTZ00335), tubulin beta chain (PLN0020), S10 plectin (pfam03501), myosin tail (cl37647), troponin (pfam00992), B41 (smart00295), and spectrin (cd00176) are also present among the 110 shared domains. The glyceraldehyde 3-phosphate dehydrogenase (GAPDH) domain (cl30355)-containing protein is also present in both proteomes.

A recent immunoaffinity capture study [29] reported that the ribosomal and cytoskeletal proteins are not part of the exosomal fraction and are likely to co-purify with the vesicular preparations. Furthermore, metabolic enzymes like GAPDH and cytosolic proteins like HSP90 are present in the non-vesicular fractions [29]. However, other reports suggest that cytoskeletal proteins are found in microvesicles [31]. Whether the abundance of ribosomal, mitochondrial, and cytoskeletal proteins in wasp venom particles is due to co-purification, or if they are part of the particles’ structures remains to be determined. The presence of an ANXA1 homolog in the proteomes of both wasps strongly suggests that the particles are more like microvesicles than exosomes, but each proteome also contains one, albeit different, exosomal marker. Further experimental characterization in these two species and additional *Leptopilina* species should help address these questions.

#### 3.2.3. Species-Unique Domains

We identified many domains specific to each proteome (Table S1). PHA02927 (cl33700, four proteins) was found to be one of the most abundant conserved domains present only in *Lb*. The serpin superfamily (cl38926, one protein) and serpin42Da-like domain (cd19601, two proteins) were found only in *Lb*. Whereas the PHA02927 superfamily domain is related to the secreted complement-binding protein, the serpins regulate protease activity and are discussed in more detail in the next section. *Lb* particles appear to lack the GTPase-like proteins previously identified in *Lh* particles [16]. Other noteworthy *Lh*-specific proteins are as follows: (a) an abundant *Lh* protein, SSp40, that is structurally similar to IpaD/SipD proteins of the Type 3 secretion systems of Gram-negative bacteria [16]; and (b) a protein with a ClyA-like superfamily domain (cl45899), a family of pore-forming toxins.

The searches for *Lb* sequences compared against the Viridae protein database identified one protein (Lb_LbFV_ORF85) that matched a *Drosophila* filamentous virus protein, annotated as Ac81-like (also identified as such by Di Giovanni et al. [34]). Ac81 is involved in the nucleocapsid envelopment of *Autographa californica* nuclear polyhedrosis virus, a highly pathogenic baculovirus that targets insects [69].

### 3.3. Many Toxins Are Present in Both Proteomes

#### 3.3.1. Toxin-like Domains Are Present in Both Venom Particle Proteomes

**Figure 3:**
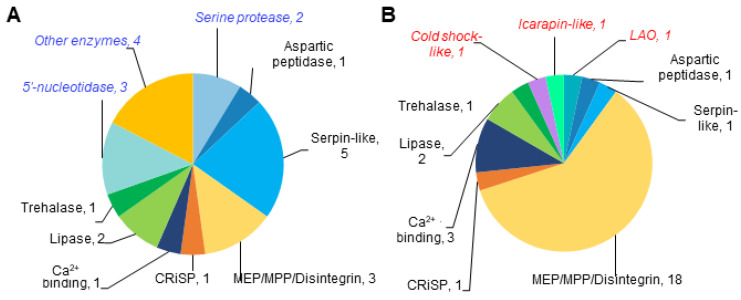
Pie charts showing the proportions of various classes of toxin-related protein domains. (**A**,**B**) The data presented are from 23 *Lb* (**A**) and 30 *Lh* (**B**) proteins. Seven toxin categories common to both species are shown in the same color. Species-specific toxin categories are italicized. The number of proteins in each category is indicated. See Tables S2 and S3 for more details.

We next asked if the proteins of the *Lb* and *Lh* venom particles share a sequence similarity to the venom proteins or toxins characterized in other taxa. We queried sequences from both wasp proteomes with the manually curated proteins with a known toxin or venom association. When using the default parameters (0.05 E-value cutoff), 46 *Lb* and 55 *Lh* proteins aligned with 272 and 293 toxin proteins, respectively. However, at the more stringent setting of 1 × 10^−5^ or less, only 23 *Lb* and *3*0 *Lh* proteins aligned with the toxin database, and these higher confidence results are presented in Figure 3, and Tables S2 and S3. We observed a high degree of correspondence in the CDD domains of the wasp query sequence, and the non-wasp toxin sequence identified (Tables S2 and S3). The proteins in several toxin categories are present in both proteomes, although their relative distribution varied. For example, while serpins and serpin-like proteins are most abundant in the *Lb* proteome, the MEP/MPP/disintegrin class dominates the toxin class in the *Lh* proteome (Figure 3). None of the putative wasp toxins are expected to be membrane-associated. Four proteins in *Lb* and fourteen in *Lh* are predicted to be secreted (Tables S2 and S3). How these putative secreted proteins become a part of the vesicle cargo remains unclear. 

The venom proteome of many animals contains proteins rich in cysteine residues [70]. To determine which *Lb* and *Lh* proteins are cysteine-rich, we scored the number of cysteine residues and identified one *Lb* and one *Lh* serpin-like protein in each proteome, with a trypsin inhibitor-like cysteine-rich domain. The two proteins contain more than 10% cysteine residues: *Lb* g1397.t1 has 15 cysteine residues out of 146 amino acids, and the *Lh* GAJC01002499.1_10 protein has 21 cysteine residues out of 168 amino acids (Tables S2 and S3). Two other *Lh* proteins, diedel-like (GAJC01010415.1_14) and LhKnot (GAJC01011813.1_4), although not identified in the toxin search, are also cysteine-rich. LhKnot contains the core knottin fold and also possesses the Cation–Polar–Cation clip [71].

For serpins, four of the five *Lb* serpins identified in the toxin search are similar to iripin-3, which, in ticks, has been found to inhibit host inflammation [72]. The last *Lb* serpin (g1397.t1) is similar to the cysteine-rich venom protein 6 from *Pimpla hypochondriaca* [73]. One *Lh* serpin (GAJC01002499.1_10) is similar to the scorpion venom peptides CtAPI and SjAPI [74].

For MEP/MPP/disintegrin, one *Lb* protein (g25658.t1) and seventeen *Lh* proteins are similar to neprilysin-1 (cl14813), which is found in spider venom [75]. Two *Lb* proteins (g23282.t1 and g8353.t1), and one *Lh* protein GAJC01029468.1_54) are similar to a snake metalloprotease/disintegrin protein and venom metalloproteinase 3 proteins, which are abundant in the pit viper venom [76]. The same three proteins are also similar to a metalloprotease from an ectoparasitoid wasp venom protein that is toxic to its host [77]. These putative *Lb* and *Lh* MEP/MPP/disintegrins constitute an interesting class of proteins worthy of future bioinformatic and experimental characterization.

For CRiSP, one protein in each wasp’s proteome (g882.t1 and GAJC01009318.1_8) contains the cl00133 CAP (cd05380 CAP_euk superfamily; cysteine-rich secretory proteins, antigen 5, and pathogenesis-related 1 proteins) domain. Some toxins with this domain, e.g., the venom allergen 3 or venom allergen 5, are major allergens to humans [78]. One CRiSP toxin, the cysteine-rich venom protein helothermine from the Mexican beaded lizard *Heloderma horridum*, can inhibit ion channels [79].

For Ca^2+^ binding, one *Lb* (g699.t1) and three *Lh* (GAJC01010984.1_6, GAJC01011653.1_5, and GAJC01013138.1_9) proteins contain the Ca^2+^-binding EF-hand superfamily domain, cl34916. This domain is also found in the snake venom calglandulin, where it appears to regulate venom secretion via Ca^2+^-mediated activities [80].

For lipase, two proteins in each proteome (*Lb*: g13378.t1 and g20934.t1; *Lh*: GAJC01027753.1_25 and GAJC01017199.1_29) contain the cl28691 triacylglycerol lipase superfamily domain, with high confidence homology to a rattlesnake venom lysosomal acid lipase/cholesteryl ester hydrolase [81].

For trehalase, one protein from each proteome (g6244.t1 and GAJC01021538.1_51) contains the trehalase domain. These proteins are similar to the parasitoid *Pimpla hypochondriaca* trehalase [82].

For aspartic peptidase, one protein in each wasp (g2172.t1 and GAJC01012383.1_11) is similar to the viper *Echis ocellatus* renin [83]. Renin is an aspartic protease that cleaves angiotensinogen into angiotensin I, which is involved in vasoconstriction in humans.

#### 3.3.2. Species-Unique Toxin-like Domains

Of the 23 toxin-like *Lb* proteins, we identified 9 proteins with domains that were not present in the *Lh* proteome. Two *Lb* proteins are serine proteases: g3398.t1 is similar to a moth hemolin. The moth protein can trigger hemorrhagic syndrome in humans [84,85]. Additionally, g2472.t1 is similar to the anticoagulant, lactotransferrin, in vampire bats [86]. Three L*b* proteins (g9134.t1, g6755.t1, and g24769.t1) are similar to the snake venom 5′-nucleotidase toxins that serve to inhibit platelet aggregation and enhance the venom’s anticoagulant effects [87].

Four additional *Lb* proteins, with domains not identified in the *Lh* proteome include the following: (a) g4648.t1: a histidine phosphatase, similar to *A. mellifera* venom acid phosphatase Acph-1, [88,89]; (b) g3260.t1: a thioredoxin-like protein, similar to the western diamondback rattlesnake Peroxiredoxin-4 [90]; (c) g14621.t1: a serine carboxypeptidase, similar to the honeybee venom serine carboxypeptidase; and (d) g1968.t1: an α/β hydrolase, similar to the honeybee venom dipeptidyl-peptidase 4 [91].

Among the 30 toxin-like *Lh* proteins, we found 3 proteins whose domains were not found in the *Lb* proteome. The *Lh* protein, GAJC01010465.1_24, contains the L-amino-acid oxidase (LAO) domain, also found in the Eastern diamondback rattlesnake venom [92]. LAOs raise ROS levels by producing hydrogen peroxide as a byproduct of their enzymatic reaction and can be involved in apoptosis. An LAO-containing Apoptosis-Inducing protein, AIP, can induce apoptosis in vitro [93]. In addition, the GAJC01009588.1_5 sequence is similar to the *E. coli* cold shock-like protein, Cspd, which inhibits DNA replication and is regulated by the toxin–antitoxin pair MsqR/MsqA [94,95]. GAJC01009987.1_9 is similar to the Icarapin and Icarapin-like protein, a bee venom allergen [96].

Thus, *Lb* and *Lh* venom particles contain a surprising variety of toxin-like biochemical activities, whose homologs are present in organisms ranging from bacteria to snakes. Why there might be so many different toxin types in parasitoid wasp venom, where these proteins are active—in the parasite or in the host—and if they target different host tissues and cells remains to be explored.

### 3.4. Bacterial ART-like Proteins Are Present in the Venom Particle Proteomes

Our pLM-BLAST [50] searches of the protein sequences lacking conserved domains revealed remote homologies and identified 44 putative ADP-ribosyltransferase (ART) enzymes, with more than 20 potential ARTs in *Lb* and *Lh* each. Found in all life forms, the ADP ribosylation system is an ancient post-translational mechanism with important roles in many cellular processes including apoptosis, cell division, DNA-damage repair, and transcription [97]. Physiologically, ARTs play a role in the hosts’ immune response to pathogenic bacteria and viruses [97]; they also serve as bacterial toxins by covalently modifying host proteins or by mimicking the functions of host proteins [62,98,99].

ARTs catalyze the substrate-specific transfer of an ADP-ribose group from NAD^+^ to a target protein to generate an ADP-ribosylated protein and nicotinamide. Being bulky and negatively charged, the ADP-ribose moiety can sterically block interactions with partner molecules, induce conformational changes, or create docking sites for new interaction partners [100]. Most ARTs use specific amino acids to interact with NAD^+^, either via the R-S-E or via the H-Y-D residues, represented, respectively, by the cholera or the diphtheria toxins [97,101].

To corroborate our initial findings, we performed DALI searches to identify the structural matches of the >40 ART-like sequences identified by pLM-BLAST for *Lb* and *Lh*. We found that 19 *Lb* and 17 *Lh* sequences have structural similarity to bacterial ARTs; several structural matches of these 36 wasp proteins belong to the R-S-E class of bacterial ARTs (e.g., Vis, VIP2, Iota, and other C3-like toxins) [62].

**Figure 4:**
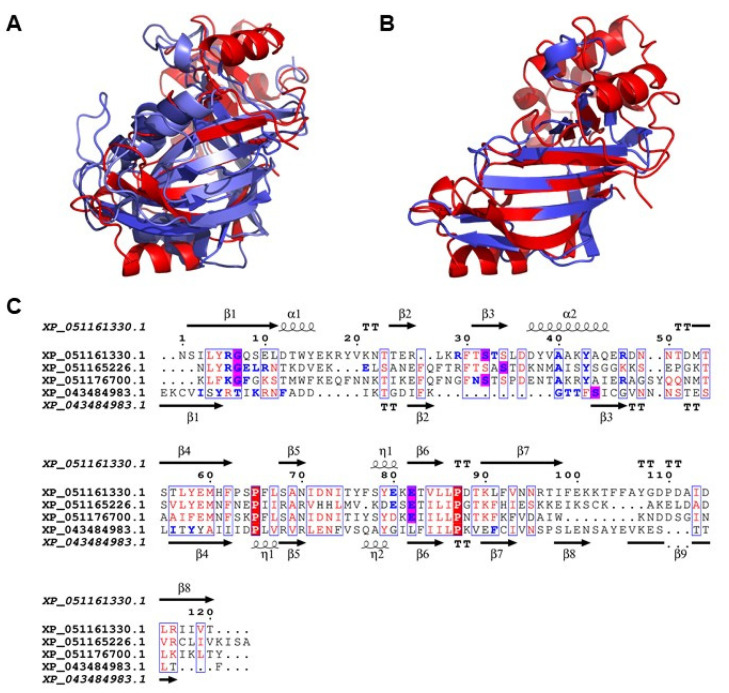
A conserved ART fold and R-S-E motif in wasp sequences. (**A**) Structural superposition of *Lb_284* (XP_051165226.1; tv_blue; RMSD: 1.84 Å)*, Lb_316* (XP_051176700.1; slate blue; RMSD: 1.61Å), and *Lb_340* (XP_051161330.1; marine blue; RMSD: 1.46 Å) with *Bacillus cereus* C3 exoenzyme (PDB: 4XSH; red). (**B**) Structural superposition of *Lh_005* (XP_043484983.1; slate blue; RMSD: 2.40 Å) with *Bacillus cereus* C3 exoenzyme (PDB: 4XSH; red). (**C**) Multiple sequence alignment of three *Lb* and one *Lh* putative ARTs visualized in ESPript. Residues shaded red denote identity matches while residues colored red and boxed in blue show similarity matches. Residues colored blue represent NAD^+^ -interacting residues and those that are shaded in magenta show NAD^+^-interacting residues that form a putative R-S-E motif. The secondary structure descriptions for the *Lb* sequences are shown above and those for the *Lh* sequence are shown below the alignment. See Table S4 and Figures S2–S5.

In the known bacterial ARTs, the R-S-E residues are located on the β-strands of the core structure, with NAD^+^ binding to the cleft formed by the core structure [62]. As seen in Figure 4, the four wasp sequences show the expected conservation of the core tertiary fold and the presence of a putative R-S-E motif. A structural superposition of a known bacterial ART, the *Bacillus cereus* C3 exoenzyme, with the three *Lb* sequences (Figure 4A) and the single *Lh* sequence (Figure 4B), shows low RMSD values ranging from 1.46 to 2.40 Å. Thus, despite a lack of sequence conservation, there is a clear similarity in the tertiary structural folds between the bacterial and the wasp proteins. The C3-like exoenzymes catalyze the transfer of the ADP-ribose moiety from the co-substrate NAD^+^ to their substrate proteins Rho A, B, and C of the Rho family of low molecular mass GTPases, rendering them inactive [63].

Additional bioinformatic analyses revealed the presence of a putative R-S-E motif in all four putative wasp ARTs. The identities of most of the R-S-E residues were validated in the docking analysis using LIGPLOTs [102] (Figure 4C and Figures S2–S5, Table S4). Although the R and E residues are present within the *Lh* motif, the docking analysis did not identify them to bind with NAD^+^. In addition, one of the three *Lb* sequences shows the presence of a K residue instead of the canonical R residue in the consensus motif. Nevertheless, the structures of all four wasp sequences exhibit a conservation of the expected core structure present in bacterial ARTs, consisting of six β-strands and a helix (Figure 4).

Our results demonstrate that combining the different available bioinformatic approaches can allow functional annotations of proteins with evidence of activities, even in sequences that fail to identify conserved domains. Additional effort is required to characterize the remaining 32 putative ART-like sequences.

Together, these results reinforce the idea that the venom particles are most similar to eukaryotic microvesicle-like extracellular vesicles. The genes encoding the constituent proteins, including the ones without apparent conserved domains, are present in genomic assemblies and are predicted to be eukaryotic in structure and contain exons and introns ([17]; our unpublished results). These findings further strengthen previous interpretations that these venom particles are similar to the organelles that assemble in venom glands and serve to transfer parasite proteins into hosts [16]. Similar studies from additional *Leptopilina* species using advanced purification techniques and a targeted biochemical analysis will enhance our understanding of their core constituents and the potential biological effects in *Drosophila* hosts. Genetic approaches in *D. melanogaster* offer great untapped potential to understand the cell biology of this class of venom particles in their natural hosts.

Even though our results are based on bioinformatic analyses from just two *Leptopilina* species, they have considerable predictive value. The venom particles from both *Leptopilina* wasps are expected to contain a striking diversity of animal and bacterial toxin-like proteins, active in the venom of other species. It is unclear why the wasp venom particles contain so many different types of toxins and this diversity may reflect the wasps’ adaptation to various hosts from different geographic regions and/or point to their evolutionary origins. It is possible that these same toxins are also present in the wasps’ venom fluid but that packaging these enzymes into subcellular entities restrains their activity until they enter their intended target cells in the host. The particles may also provide a way to condense key effectors to ensure that they achieve threshold concentrations in the target cells. The particles themselves are internalized into plasmatocytes and lamellocytes via distinct cellular mechanisms. Once inside host cells, the fate of the particles and the fates of the target cells vary depending on the hemocyte type [27]. It is likely that the particle proteins themselves mediate hemocyte specificity and trigger downstream events.

Although the venom and venom proteins from animals have been characterized, there are only a few reports of EVs in venom (e.g., [103]). If venom EVs help the bioactive proteins to reach their correct cellular targets and concentrate effector proteins for optimal activity, as we propose, then similar vesicles should be commonplace in the venom secretions of other invertebrate and vertebrate animals. Furthermore, the use of EVs to deliver toxins may be a common theme in host–parasite/predator–prey interactions.

EVs have been identified in all three domains of life [104,105]. Their composition and diversity are not as well understood in insects as they are in mammals. Our studies show shared properties between insect proteomes and human EVs. Future studies from different insect species will reveal the diversity of particle types and their protein constituents. These studies should highlight the cell-biological features and evolutionary profiles unique to insect EVs. It has been proposed that the origin of the first cell may be linked to EVs or viral origins [106]. Thus, deeper insights into wasp venom particles will reveal their relationship with their cells of origin and their target cells in the context of predator-to-prey relationships. EVs are also associated with numerous pathologies [67] and their analysis in diverse biological systems can provide therapeutic avenues. Knowledge of their protein constituents, including the putative ARTs identified in this study, will help develop novel immune therapies and ways to control insect pests.

## 4. Conclusions

Our side-by-side examination of the venom glands and venom particles from two *Leptopilina* species, from different clades, share essential features in their proteomes, which greatly simplifies our view of their identities and the possible functional activities they carry. They also raise new questions. Our key findings are as follows: (a) The wasps have a similar venom gland organ architecture. (b) The overall composition of the proteomes is similar; both contain many more proteins with conserved domains than proteins lacking conserved domains. The proteins in the latter category are very likely to be critical in defining differences in the venom activities of the two wasps. Where conserved domains are present, there are also key differences in the number of proteins represented. Members of the GluZincin superfamily stand out in this regard. (c) A vast majority of the proteins are predicted to be cytoplasmic. (d) There is a strong enrichment of the endomembrane system and exosomal proteins, especially if the over-representation of ribosomal, mitochondrial, and metabolic enzymes is due to the co-purification of these structures or their high abundance in eukaryotic cells. (e) One Annexin A1 homolog, a marker for human microvesicles, is present in each proteome. (f) Many proteins known to act as toxins in other organisms are associated with the venom particles. (g) Proteins lacking conserved domains can be understood by utilizing AlphaFold and other sensitive bioinformatic tools. The novel proteins predicted to contain bacterial ART-like folds are present in the particles. (h) While many species-specific proteins are present in the proteomes, no distinct trends were apparent. (i) Both proteomes lack clear-cut viral proteins.

## Data Availability

The *Leptopilina heterotoma* sequences are publicly available (NCBI Accession: GAJC00000000.1). The *Leptopilina boulardi* sequences are available upon request.

## Supplementary Materials

The following supporting information can be downloaded at https://www.mdpi.com/article/10.3390/pathogens14030255/s1: Figure S1. Multiple sequence alignment of human Annexin A1 sequence with homologs from the *L. boulardi* and *L. heterotoma* venom particle proteomes. Figures S2–S5. Secondary structure plots and LIGPLOTs of ART-like sequences. Table S1. Conserved domains in proteins found in the *L. boulardi* and *L. heterotoma* venom particles. Table S2. *Leptopilina boulardi* proteins with high confidence homology to toxins. Table S3. *Leptopilina heterotoma* proteins with high confidence homology to toxins. Table S4. Bacterial ADP-ribosyltransferase-like proteins from wasp venom particles.

## References

[1] Burke G.R., Sharanowski B.J. (2024). Parasitoid wasps. Curr. Biol..

[2] Lue C.H., Buffington M.L., Scheffer S., Lewis M., Elliot T.A., Lindsey A.R.I., Driskell A., Jandova A., Kimura M.T., Carton Y. (2021). DROP: Molecular voucher database for identification of *Drosophila* parasitoids. Mol. Ecol. Resour..

[3] Kim-Jo C., Gatti J.L., Poirié M. (2019). *Drosophila* cellular immunity against parasitoid wasps: A complex and time-dependent process. Front. Physiol..

[4] Nappi A., Poirié M., Carton Y. (2009). The role of melanization and cytotoxic side-products in the cellular immune responses of Drosophila against parasitic wasps. Adv. Parasitol..

[5] Salvia R., Cozzolino F., Scieuzo C., Grimaldi A., Franco A., Vinson S.B., Monti M., Falabella P. (2022). Identification and functional characterization of *Toxoneuron nigriceps* ovarian proteins involved in the early suppression of host immune response. Insects.

[6] Strand M.R., Burke G.R. (2020). Polydnaviruses: Evolution and function. Curr. Issues Mol. Biol..

[7] Rizki R.M., Rizki T.M. (1984). Selective destruction of a host blood cell type by a parasitoid wasp. Proc. Natl. Acad. Sci. USA.

[8] Moreau S.J.M., Asgari S. (2015). Venom proteins from parasitoid wasps and their biological functions. Toxins.

[9] Volkoff A.-N., Cusson M. (2020). The unconventional viruses of ichneumonid parasitoid wasps. Viruses.

[10] Drezen J.-M., Bézier A., Burke G.R., Strand M.R. (2022). Bracoviruses, ichnoviruses, and virus-like particles from parasitoid wasps retain many features of their virus ancestors. Curr. Opin. Insect Sci..

[11] Quicke D.L.J., Butcher B.A. (2021). Review of venoms of non-polydnavirus carrying ichneumonoid wasps. Biology.

[12] Heavner M.E., Hudgins A.D., Rajwani R., Morales J., Govind S. (2014). Harnessing the natural *Drosophila*-parasitoid model for integrating insect immunity with functional venomics. Curr. Opin. Insect Sci..

[13] Poirié M., Colinet D., Gatti J.-L. (2014). Insights into function and evolution of parasitoid wasp venoms. Curr. Opin. Insect Sci..

[14] Schlenke T.A., Morales J., Govind S., Clark A.G. (2007). Contrasting infection strategies in generalist and specialist wasp parasitoids of *Drosophila melanogaster*. PLoS Pathog..

[15] Wan B., Goguet E., Ravallec M., Pierre O., Lemauf S., Volkoff A.-N., Gatti J.-L., Poirié M. (2019). Venom atypical extracellular vesicles as interspecies vehicles of virulence factors involved in host specificity: The case of a *Drosophila* parasitoid wasp. Front. Immunol..

[16] Heavner M.E., Ramroop J., Gueguen G., Ramrattan G., Dolios G., Scarpati M., Kwiat J., Bhattacharya S., Wang R., Singh S. (2017). Novel organelles with elements of bacterial and eukaryotic secretion systems weaponize parasites of *Drosophila*. Curr. Biol..

[17] Wey B., Heavner M.E., Wittmeyer K.T., Briese T., Hopper K.R., Govind S. (2020). Immune suppressive extracellular vesicle proteins of *Leptopilina heterotoma* are encoded in the wasp genome. G3.

[18] Rizki R.M., Rizki T.M. (1990). Parasitoid virus-like particles destroy *Drosophila* cellular immunity. Proc. Natl. Acad. Sci. USA.

[19] Gueguen G., Rajwani R., Paddibhatla I., Morales J., Govind S. (2011). VLPs of *Leptopilina boulardi* share biogenesis and overall stellate morphology with VLPs of the *heterotoma* clade. Virus Res..

[20] Chiu H., Morales J., Govind S. (2006). Identification and immuno-electron microscopy localization of p40, a protein component of immunosuppressive virus-like particles from *Leptopilina heterotoma*, a virulent parasitoid wasp of *Drosophila*. J. Gen. Virol..

[21] Rizki T.M., Rizki R.M. (1994). Parasitoid-induced cellular immune deficiency in *Drosophila*. Ann. N. Y Acad. Sci..

[22] Wey B. (2021). Insights into *Leptopilina* spp. immune-suppressive strategies using mixed-omics and molecular approaches. Ph.D. Thesis.

[23] Colinet D., Schmitz A., Depoix D., Crochard D., Poirié M. (2007). Convergent use of RhoGAP toxins by eukaryotic parasites and bacterial pathogens. PLoS Pathog..

[24] Wan B., Poirié M., Gatti J.-L. (2020). Parasitoid wasp venom vesicles (venosomes) enter *Drosophila melanogaster* lamellocytes through a flotillin/lipid raft-dependent endocytic pathway. Virulence.

[25] Colinet D., Schmitz A., Cazes D., Gatti J.-L., Poirié M. (2010). The origin of intraspecific variation of virulence in an eukaryotic immune suppressive parasite. PLoS Pathog..

[26] Chiu H., Govind S. (2002). Natural infection of *D. melanogaster* by virulent parasitic wasps induces apoptotic depletion of hematopoietic precursors. Cell Death Differ..

[27] Ramroop J.R., Heavner M.E., Razzak Z.H., Govind S. (2021). A parasitoid wasp of *Drosophila* employs preemptive and reactive strategies to deplete its host’s blood cells. PLoS Pathog..

[28] Beer K.B., Wehman A.M. (2017). Mechanisms and functions of extracellular vesicle release in vivo—What we can learn from flies and worms. Cell Adh Migr..

[29] Jeppesen D.K., Fenix A.M., Franklin J.L., Higginbotham J.N., Zhang Q., Zimmerman L.J., Liebler D.C., Ping J., Liu Q., Evans R. (2019). Reassessment of exosome composition. Cell.

[30] van Niel G., D’Angelo G., Raposo G. (2018). Shedding light on the cell biology of extracellular vesicles. Nat. Rev. Mol. Cell Biol..

[31] Lim H.J., Yoon H., Kim H., Kang Y.-W., Kim J.-E., Kim O.Y., Lee E.-Y., Twizere J.-C., Rak J., Kim D.-K. (2021). Extracellular vesicle proteomes shed light on the evolutionary, interactive, and functional divergence of their biogenesis mechanisms. Front. Cell Dev. Biol..

[32] Heavner M.E., Gueguen G., Rajwani R., Pagan P.E., Small C., Govind S. (2013). Partial venom gland transcriptome of a *Drosophila* parasitoid wasp, *Leptopilina heterotoma*, reveals novel and shared bioactive profiles with stinging Hymenoptera. Gene.

[33] Small C., Paddibhatla I., Rajwani R., Govind S. (2012). An introduction to parasitic wasps of *Drosophila* and the antiparasite immune response. J. Vis. Exp..

[34] Di Giovanni D., Lepetit D., Guinet B., Bennetot B., Boulesteix M., Couté Y., Bouchez O., Ravallec M., Varaldi J. (2020). A behavior-manipulating virus relative as a source of adaptive genes for *Drosophila* parasitoids. Mol. Biol. Evol..

[35] Goecks J., Mortimer N.T., Mobley J.A., Bowersock G.J., Taylor J., Schlenke T.A. (2013). Integrative approach reveals composition of endoparasitoid wasp venoms. PLoS ONE.

[36] Käll L., Krogh A., Sonnhammer E.L.L. (2004). A combined transmembrane topology and signal peptide prediction method. J. Mol. Biol..

[37] Käll L., Krogh A., Sonnhammer E.L.L. (2007). Advantages of combined transmembrane topology and signal peptide prediction—The Phobius web server. Nucleic Acids Res..

[38] Nielsen H. (2017). Predicting secretory proteins with SignalP. Methods Mol. Biol..

[39] Almagro Armenteros J.J., Tsirigos K.D., Sønderby C.K., Petersen T.N., Winther O., Brunak S., von Heijne G., Nielsen H. (2019). SignalP 5.0 improves signal peptide predictions using deep neural networks. Nat. Biotechnol..

[40] Sonnhammer E.L.L., von Heijne G., Krogh A. (1998). A hidden Markov model for predicting transmembrane helices in protein sequences. Proc. Int. Conf. Intell. Syst. Mol. Biol..

[41] Krogh A., Larsson B., von Heijne G., Sonnhammer E.L.L. (2001). Predicting transmembrane protein topology with a hidden Markov model: Application to complete genomes. J. Mol. Biol..

[42] Savojardo C., Bruciaferri N., Tartari G., Martelli P.L., Casadio R. (2019). DeepMito: Accurate prediction of protein sub-mitochondrial localization using convolutional neural networks. Bioinformatics.

[43] Kalra H., Simpson R.J., Ji H., Aikawa E., Altevogt P., Askenase P., Bond V.C., Borràs F.E., Breakefield X., Budnik V. (2012). Vesiclepedia: A compendium for extracellular vesicles with continuous community annotation. PLoS Biol..

[44] Pathan M., Fonseka P., Chitti S.V., Kang T., Sanwlani R., Van Deun J., Hendrix A., Mathivanan S. (2019). Vesiclepedia 2019: A compendium of RNA, proteins, lipids and metabolites in extracellular vesicles. Nucleic Acids Res..

[45] Pathan M., Keerthikumar S., Chisanga D., Alessandro R., Ang C.-S., Askenase P., Batagov A.O., Benito-Martin A., Camussi G., Clayton A. (2017). A novel community driven software for functional enrichment analysis of extracellular vesicles data. J. Extracell. Vesicles.

[46] Di Tommaso P., Moretti S., Xenarios I., Orobitg M., Montanyola A., Chang J.-M., Taly J.-F., Notredame C. (2011). T-Coffee: A web server for the multiple sequence alignment of protein and RNA sequences using structural information and homology extension. Nucleic Acids Res..

[47] Robert X., Gouet P. (2014). Deciphering key features in protein structures with the new ENDscript server. Nucleic Acids Res..

[48] Jungo F., Bairoch A. (2005). Tox-Prot, the toxin protein annotation program of the Swiss-Prot protein knowledgebase. Toxicon.

[49] Jungo F., Bougueleret L., Xenarios I., Poux S. (2012). The UniProtKB/Swiss-Prot Tox-Prot program: A central hub of integrated venom protein data. Toxicon.

[50] Kaminski K., Ludwiczak J., Pawlicki K., Alva V., Dunin-Horkawicz S. (2023). pLM-BLAST: Distant homology detection based on direct comparison of sequence representations from protein language models. Bioinformatics.

[51] Huang J., Chen J., Fang G., Pang L., Zhou S., Zhou Y., Pan Z., Zhang Q., Sheng Y., Lu Y. (2021). Two novel venom proteins underlie divergent parasitic strategies between a generalist and a specialist parasite. Nat. Commun..

[52] Altschul S.F., Gish W., Miller W., Myers E.W., Lipman D.J. (1990). Basic local alignment search tool. J. Mol. Biol..

[53] Johnson M., Zaretskaya I., Raytselis Y., Merezhuk Y., McGinnis S., Madden T.L. (2008). NCBI BLAST: A better web interface. Nucleic Acids Res..

[54] Camacho C., Coulouris G., Avagyan V., Ma N., Papadopoulos J., Bealer K., Madden T.L. (2009). BLAST+: Architecture and applications. BMC Bioinform..

[55] Abramson J., Adler J., Dunger J., Evans R., Green T., Pritzel A., Ronneberger O., Willmore L., Ballard A.J., Bambrick J. (2024). Accurate structure prediction of biomolecular interactions with AlphaFold 3. Nature.

[56] Holm L., Laiho A., Törönen P., Salgado M. (2023). DALI shines a light on remote homologs: One hundred discoveries. Protein Sci..

[57] Schrodinger, LLC (2010). The PyMol Molecular Graphics System, Version 3.0.2.

[58] Heinig M., Frishman D. (2004). STRIDE: A web server for secondary structure assignment from known atomic coordinates of proteins. Nucleic Acids Res..

[59] Laskowski R.A., Thornton J.M. (2022). PDBsum extras: SARS-CoV-2 and AlphaFold models. Protein Sci..

[60] Sievers F., Wilm A., Dineen D., Gibson T.J., Karplus K., Li W., Lopez R., McWilliam H., Remmert M., Söding J. (2011). Fast, scalable generation of high-quality protein multiple sequence alignments using Clustal Omega. Mol. Syst. Biol..

[61] Toda A., Tsurumura T., Yoshida T., Tsumori Y., Tsuge H. (2015). Rho GTPase recognition by C3 exoenzyme based on C3-RhoA complex structure. J. Biol. Chem..

[62] Yoshida T., Tsuge H. (2021). Common mechanism for target specificity of protein- and DNA-targeting ADP-ribosyltransferases. Toxins.

[63] Vogelsgesang M., Pautsch A., Aktories K. (2007). C3 exoenzymes, novel insights into structure and action of Rho-ADP-ribosylating toxins. Naunyn Schmiedebergs Arch. Pharmacol..

[64] Morales J., Chiu H., Oo T., Plaza R., Hoskins S., Govind S. (2005). Biogenesis, structure, and immune-suppressive effects of virus-like particles of a *Drosophila* parasitoid, *Leptopilina victoriae*. J. Insect Physiol..

[65] Ferrarese R., Morales J., Fimiarz D., Webb B.A., Govind S. (2009). A supracellular system of actin-lined canals controls biogenesis and release of virulence factors in parasitoid venom glands. J. Exp. Biol..

[66] Labrosse C., Eslin P., Doury G., Drezen J.M., Poirié M. (2005). Haemocyte changes in *D. melanogaster* in response to long gland components of the parasitoid wasp *Leptopilina boulardi*: A Rho-GAP protein as an important factor. J. Insect Physiol..

[67] Rogers M.A., Buffolo F., Schlotter F., Atkins S.K., Lee L.H., Halu A., Blaser M.C., Tsolaki E., Higashi H., Luther K. (2020). Annexin A1-dependent tethering promotes extracellular vesicle aggregation revealed with single-extracellular vesicle analysis. Sci. Adv..

[68] Skryabin G.O., Komelkov A.V., Galetsky S.A., Bagrov D.V., Evtushenko E.G., Nikishin I.I., Zhordaniia K.I., Savelyeva E.E., Akselrod M.E., Paianidi I.G. (2021). Stomatin is highly expressed in exosomes of different origin and is a promising candidate as an exosomal marker. J. Cell Biochem..

[69] Dong F., Wang J., Deng R., Wang X. (2016). *Autographa californica* multiple nucleopolyhedrovirus gene ac81 is required for nucleocapsid envelopment. Virus Res..

[70] Ho T.N.T., Turner A., Pham S.H., Nguyen H.T., Nguyen L.T.T., Nguyen L.T., Dang T.T. (2023). Cysteine-rich peptides: From bioactivity to bioinsecticide applications. Toxicon.

[71] Arguelles J., Lee J., Cardenas L.V., Govind S., Singh S. (2023). In silico analysis of a *Drosophila* parasitoid venom peptide reveals prevalence of the cation-polar-cation clip motif in knottin proteins. Pathogens.

[72] Chlastáková A., Kotál J., Beránková Z., Kaščáková B., Martins L.A., Langhansová H., Prudnikova T., Ederová M., Kutá Smatanová I., Kotsyfakis M. (2021). Iripin-3, a new salivary protein isolated from *Ixodes ricinus* ticks, displays immunomodulatory and anti-hemostatic properties in vitro. Front. Immunol..

[73] Parkinson N.M., Conyers C., Keen J., MacNicoll A., Smith I., Audsley N., Weaver R. (2004). Towards a comprehensive view of the primary structure of venom proteins from the parasitoid wasp *Pimpla hypochondriaca*. Insect Biochem. Mol. Biol..

[74] Chen Z., Wang B., Hu J., Yang W., Cao Z., Zhuo R., Li W., Wu Y. (2013). SjAPI, the first functionally characterized *Ascaris*-type protease inhibitor from animal venoms. PLoS ONE.

[75] Undheim E.A.B., Sunagar K., Herzig V., Kely L., Low D.H.W., Jackson T.N.W., Jones A., Kurniawan N., King G.F., Ali S.A. (2013). A proteomics and transcriptomics investigation of the venom from the barychelid spider *Trittame loki* (brush-foot trapdoor). Toxins.

[76] Deshimaru M., Ichihara M., Hattori T., Koba K., Terada S. (2005). Primary structure of brevilysin L4, an enzymatically active fragment of a disintegrin precursor from *Gloydius halys brevicaudus* venom. Toxicon.

[77] Price D.R.G., Bell H.A., Hinchliffe G., Fitches E., Weaver R., Gatehouse J.A. (2009). A venom metalloproteinase from the parasitic wasp *Eulophus pennicornis* is toxic towards its host, tomato moth (*Lacanobia oleracae*). Insect Mol. Biol..

[78] Pantera B., Hoffman D.R., Carresi L., Cappugi G., Turillazzi S., Manao G., Severino M., Spadolini I., Orsomando G., Moneti G. (2003). Characterization of the major allergens purified from the venom of the paper wasp *Polistes gallicus*. Biochim. et Biophys. Acta (BBA)—General. Subj..

[79] Morrissette J., Krätzschmar J., Haendler B., el-Hayek R., Mochca-Morales J., Martin B.M., Patel J.R., Moss R.L., Schleuning W.D., Coronado R. (1995). Primary structure and properties of helothermine, a peptide toxin that blocks ryanodine receptors. Biophys. J..

[80] Junqueira-de-Azevedo I.d.L.M., Pertinhez T., Spisni A., Carreño F.R., Farah C.S., Ho P.L. (2003). Cloning and expression of calglandulin, a new EF-hand protein from the venom glands of *Bothrops insularis* snake in *E. coli*. Biochim. Biophys. Acta.

[81] Corrêa-Netto C., Junqueira-de-Azevedo I.d.L.M., Silva D.A., Ho P.L., Leitao-de-Araujo M., Alves M.L., Sanz L., Foguel D., Zingali R.B., Calvete J.J. (2011). Snake venomics and venom gland transcriptomic analysis of Brazilian coral snakes, *Micrurus altirostris* and *M. corallinus*. J. Proteom..

[82] Parkinson N.M., Conyers C.M., Keen J.N., MacNicoll A.D., Smith I., Weaver R.J. (2003). cDNAs encoding large venom proteins from the parasitoid wasp *Pimpla hypochondriaca* identified by random sequence analysis. Comp. Biochem. Physiol. C Toxicol. Pharmacol..

[83] Wilkinson M.C., Nightingale D.J.H., Harrison R.A., Wagstaff S.C. (2017). Isolation and characterization of renin-like aspartic-proteases from *Echis ocellatus* venom. Toxicon.

[84] Alvarez-Flores M.P., Fritzen M., Reis C.V., Chudzinski-Tavassi A.M. (2006). Losac, a factor X activator from Lonomia obliqua bristle extract: Its role in the pathophysiological mechanisms and cell survival. Biochem. Biophys. Res. Commun..

[85] Alvarez-Flores M.P., Furlin D., Ramos O.H.P., Balan A., Konno K., Chudzinski-Tavassi A.M. (2011). Losac, the first hemolin that exhibits procogulant activity through selective factor X proteolytic activation. J. Biol. Chem..

[86] Fernandez A.Z., Tablante A., Bartoli F., Beguin S., Hemker H.C., Apitz-Castro R. (1998). Expression of biological activity of draculin, the anticoagulant factor from vampire bat saliva, is strictly dependent on the appropriate glycosylation of the native molecule. Biochim. Biophys. Acta.

[87] Rokyta D.R., Lemmon A.R., Margres M.J., Aronow K. (2012). The venom-gland transcriptome of the eastern diamondback rattlesnake (*Crotalus adamanteus*). BMC Genom..

[88] Grunwald T., Bockisch B., Spillner E., Ring J., Bredehorst R., Ollert M.W. (2006). Molecular cloning and expression in insect cells of honeybee venom allergen acid phosphatase (Api m 3). J. Allergy Clin. Immunol..

[89] Hoffman D.R., Weimer E.T., Sakell R.H., Schmidt M. (2005). Sequence and characterization of honeybee venom acid phosphatase. J. Allergy Clin. Immunol..

[90] Calvete J.J., Fasoli E., Sanz L., Boschetti E., Righetti P.G. (2009). Exploring the venom proteome of the western diamondback rattlesnake, Crotalus atrox, via snake venomics and combinatorial peptide ligand library approaches. J. Proteome Res..

[91] Blank S., Seismann H., Bockisch B., Braren I., Cifuentes L., McIntyre M., Rühl D., Ring J., Bredehorst R., Ollert M.W. (2010). Identification, recombinant expression, and characterization of the 100 kDa high molecular weight Hymenoptera venom allergens Api m 5 and Ves v 3. J. Immunol..

[92] Raibekas A.A., Massey V. (1998). Primary structure of the snake venom L-amino acid oxidase shows high homology with the mouse B cell interleukin 4-induced Fig1 protein. Biochem. Biophys. Res. Commun..

[93] Murakawa M., Jung S.K., Iijima K., Yonehara S., Apoptosis-inducing protein (2001). AIP, from parasite-infected fish induces apoptosis in mammalian cells by two different molecular mechanisms. Cell Death Differ..

[94] Kim Y., Wang X., Zhang X.-S., Grigoriu S., Page R., Peti W., Wood T.K. (2010). *Escherichia coli* toxin/antitoxin pair MqsR/MqsA regulate toxin CspD. Environ. Microbiol..

[95] Yamanaka K., Zheng W., Crooke E., Wang Y.-H., Inouye M. (2001). CspD, a novel DNA replication inhibitor induced during the stationary phase in *Escherichia coli*. Mol. Microbiol..

[96] Wong K.-L., Li H., Wong K.-K.K., Jiang T., Shaw P.-C. (2012). Location and reduction of icarapin antigenicity by site specific coupling to polyethylene glycol. Protein Pept. Lett..

[97] Mikolčević P., Hloušek-Kasun A., Ahel I., Mikoč A. (2021). ADP-ribosylation systems in bacteria and viruses. Comput. Struct. Biotechnol. J..

[98] Simon N.C., Aktories K., Barbieri J.T. (2014). Novel bacterial ADP-ribosylating toxins: Structure and function. Nat. Rev. Microbiol..

[99] Aktories K. (2011). Bacterial protein toxins that modify host regulatory GTPases. Nat. Rev. Microbiol..

[100] Laing S., Unger M., Koch-Nolte F., Haag F. (2011). ADP-ribosylation of arginine. Amino Acids.

[101] Hottiger M.O., Hassa P.O., Luscher B., Schuler H., Koch-Nolte F. (2010). Toward a unified nomenclature for mammalian ADP-ribosyltransferases. Trends Biochem. Sci..

[102] Laskowski R.A., Swindells M.B. (2011). LigPlot+: Multiple ligand-protein interaction diagrams for drug discovery. J. Chem. Inf. Model..

[103] Nagendra K., Bakkannavar S.M., Bhat V.R., Sirur F.M. (2024). A review on snake venom extracellular vesicles: Past to present. Toxicon.

[104] Gill S., Catchpole R., Forterre P. (2019). Extracellular membrane vesicles in the three domains of life and beyond. FEMS Microbiol. Rev..

[105] Rojas A., Regev-Rudzki N. (2024). Biogenesis of extracellular vesicles from the pathogen perspective: Transkingdom strategies for delivering messages. Curr. Opin. Cell Biol..

[106] Gill S., Forterre P. (2016). Origin of life: LUCA and extracellular membrane vesicles (EMVs). Int. J. Astrobiol..

