# Supplementary material for: Venomous Cargo: Diverse Toxin-Related Proteins Are Associated with Extracellular Vesicles in Parasitoid Wasp Venom
Source: Pathogens. 2025 Mar 5;14(3):255. doi: 10.3390/pathogens14030255 (PMC11944595; doi:10.3390/pathogens14030255)
Supplement: Supplementary file 1 [file pathogens-14-00255-s001.zip › pathogens-3447098-supplementary.pdf]

Supplementary Figure S1.

Multiple sequence alignment of human Annexin A1 sequence with homologs from the *L. boulardi* and *L. heterotoma* venom particle proteomes.

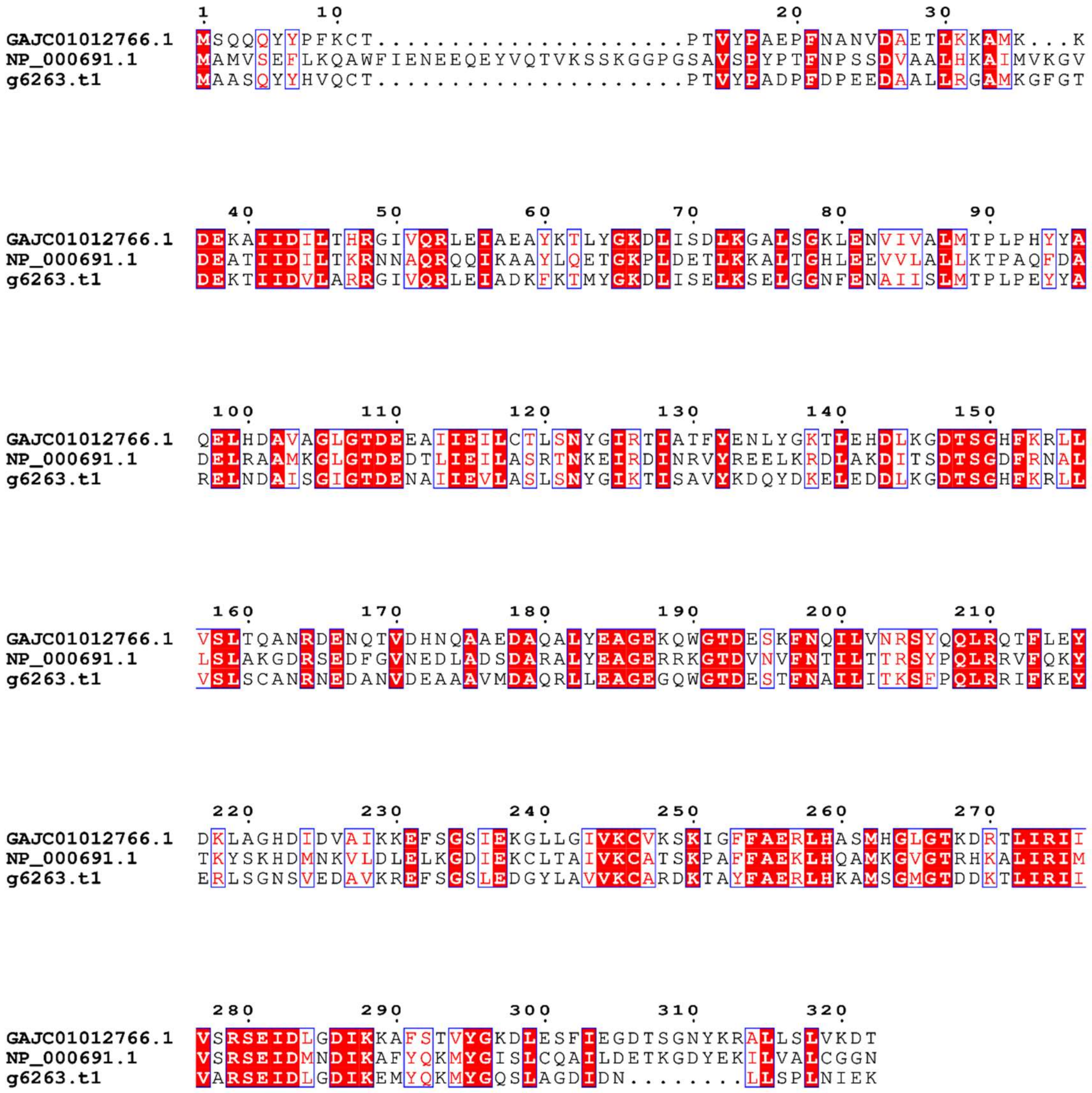

Supplementary Figures S2-S5.

Secondary structure plots and LIGPLOTs for ART-like wasp sequences. (A) The primary and secondary structures (SS) of the bacterial ADP-ribosyltransferase-like proteins from EV-like structures in wasp venom were obtained using molecular modeling software (see Methods). For each putative bacterial ART-like sequence, the presence of alpha-helices (labeled H1, H2, etc.) and beta sheets (labeled A and B) are shown. Beta and gamma turns are also labeled. Beta hairpin is shown with a red hairpin. Disulfide bridges are shown in yellow. Predicted NAD^+-interacting amino acid residues based on docking analyses (residues colored blue in the alignment of the single beta sandwich region in Figure 4C) are shown with a filled red square. **(B)** A LIGPLOT showing two-dimensional representation of the NAD^+ ligand binding to amino acid residues in the single beta sandwich region. Amino acid residues are numbered in the context of the predicted full-length protein shown in the SS plot. Ligand bond (purple), non-ligand bond (mustard), dashes (H-bond and its length), non-ligand hydrophobic interactions (eyelashes), atoms involved in hydrophobic contacts (small eyelashes) are shown. Figures are shown in the following order:

Figure S2A. SS Plot *Lb_284*; **Figure S2B.** LIGPLOT *Lb_284*
Figure S3A. SS Plot *Lb_316*; **Figure S3B.** LIGPLOT *Lb_316*
Figure S4A. SS Plot *Lb_340*; **Figure S4B.** LIGPLOT *Lb_340*
Figure S5A. SS Plot *Lh_005*; **Figure S5B.** LIGPLOT *Lh_005*

Figure S2A. SS Plot *Lb_284*

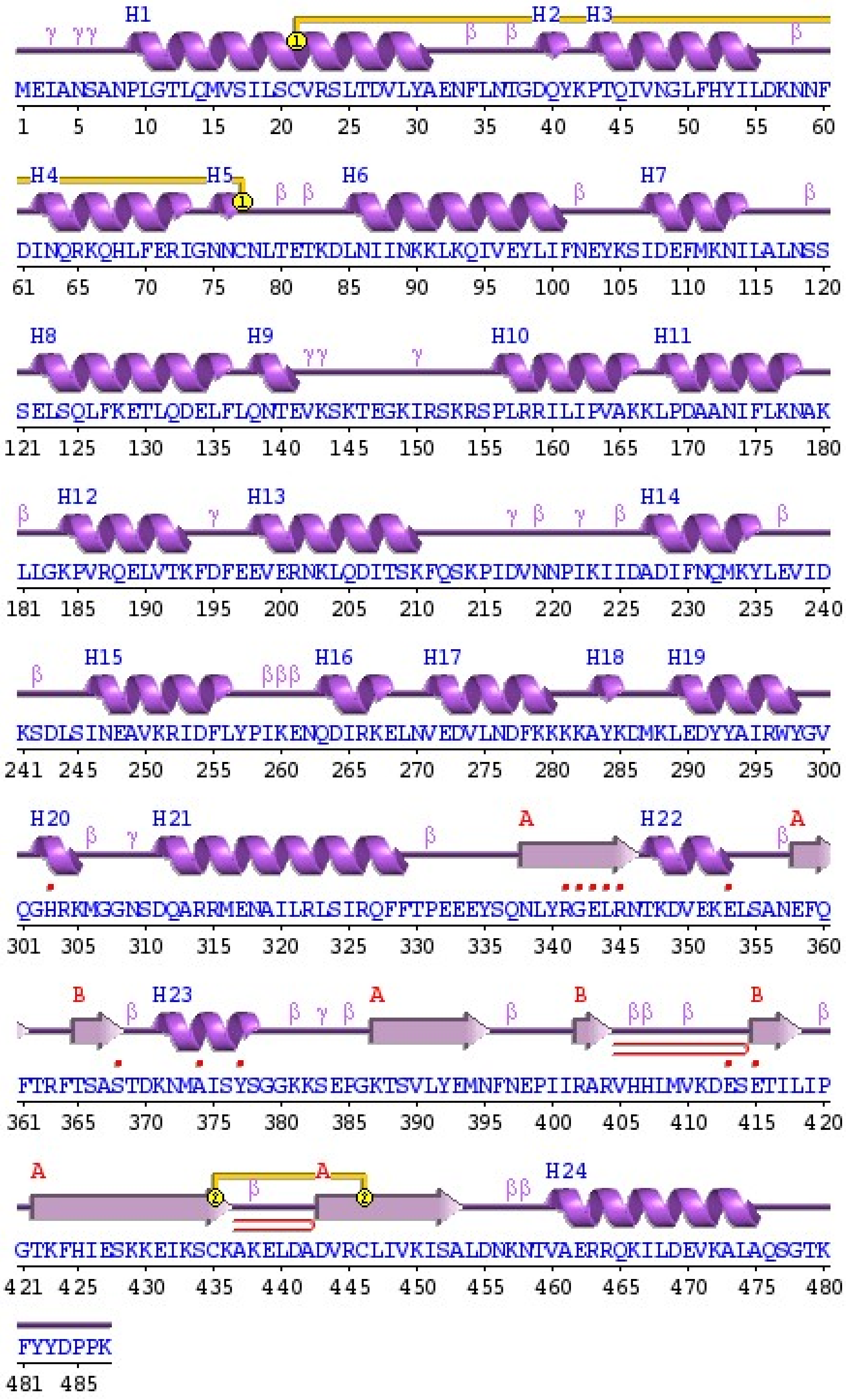

Figure S2B. LIGPLOT *Lb_284*

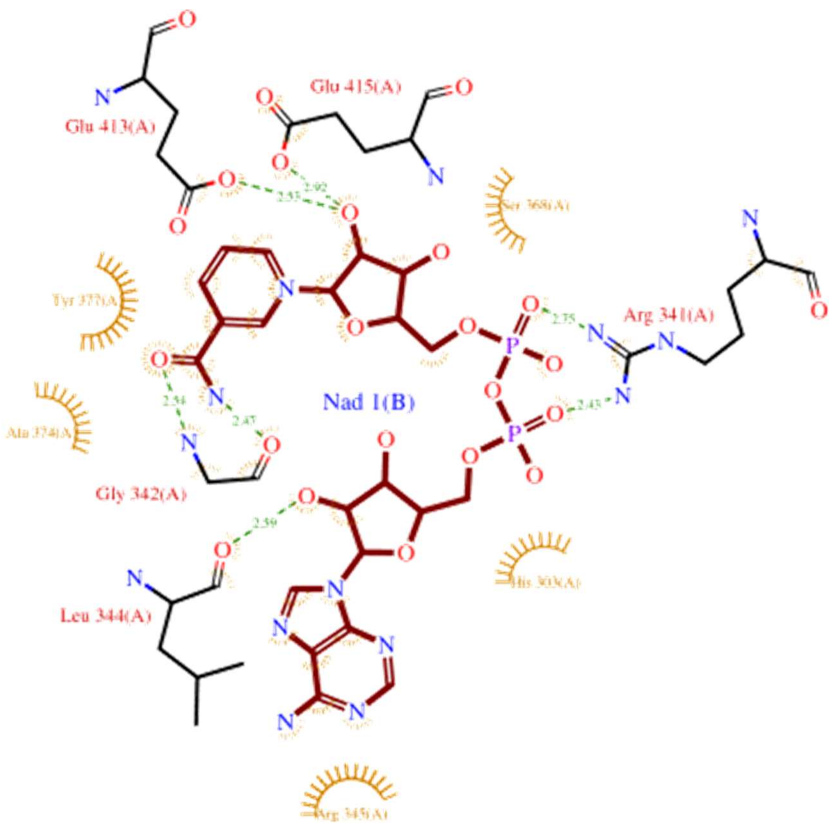

Figure S3A. SS Plot *Lb_316*

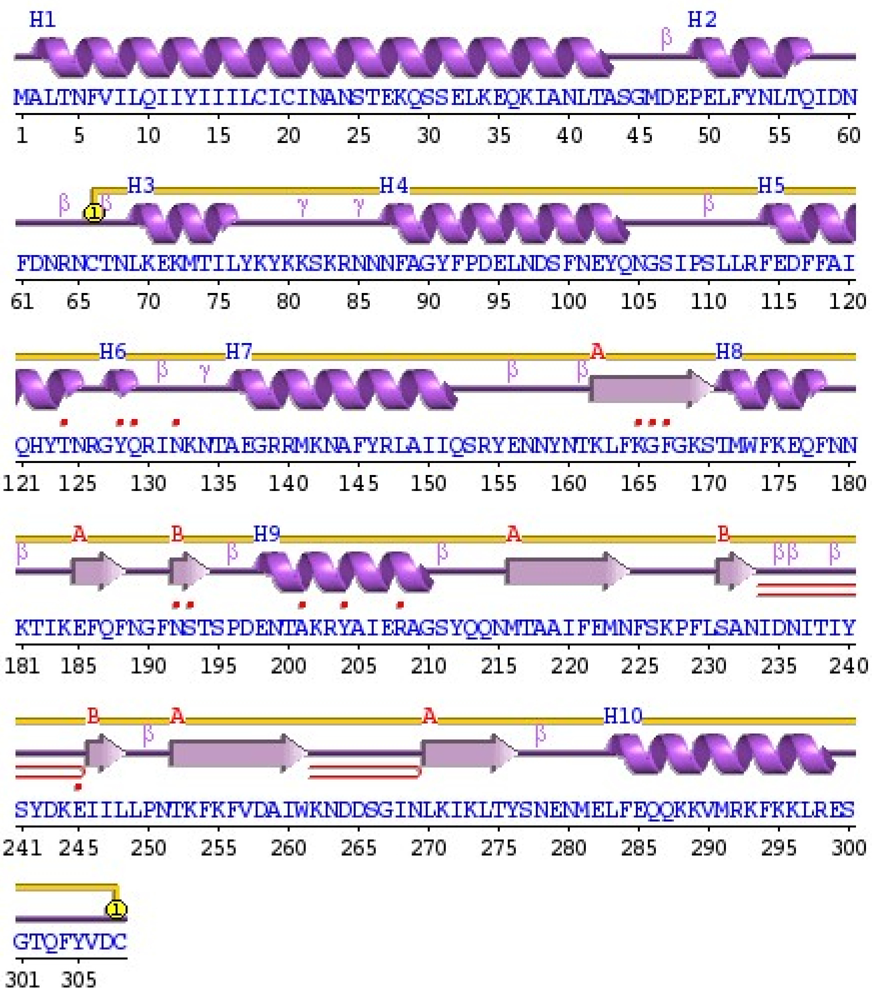

Figure S3B. LIGPLOT *Lb_316*

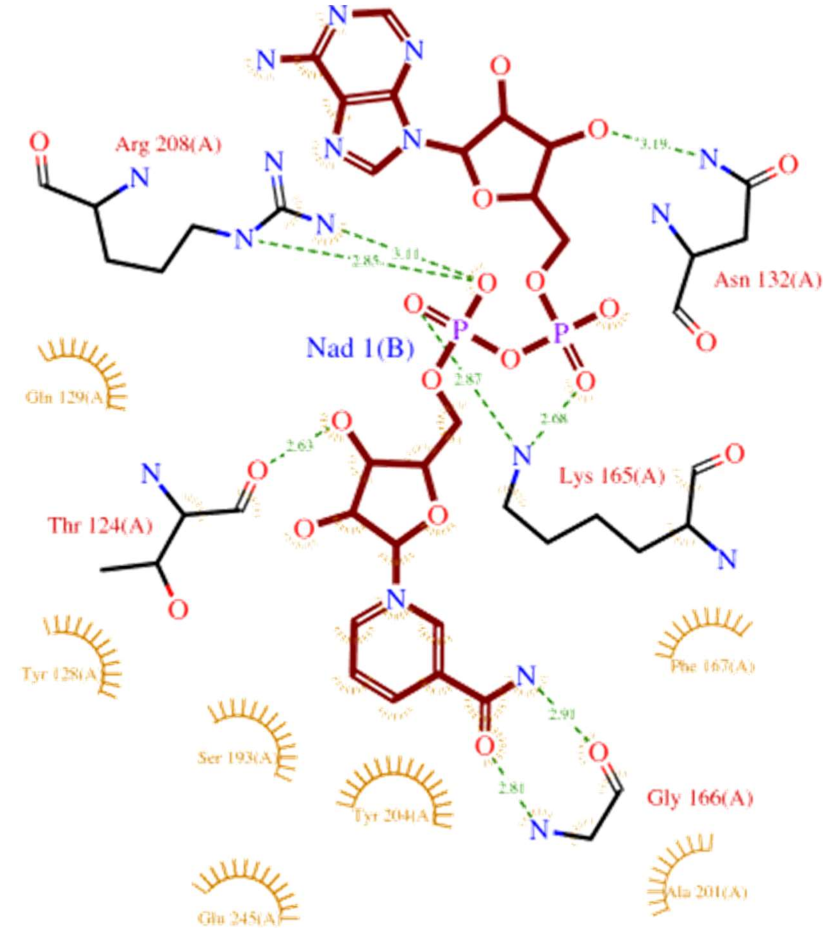

Figure S4A. SS Plot *Lb_340*

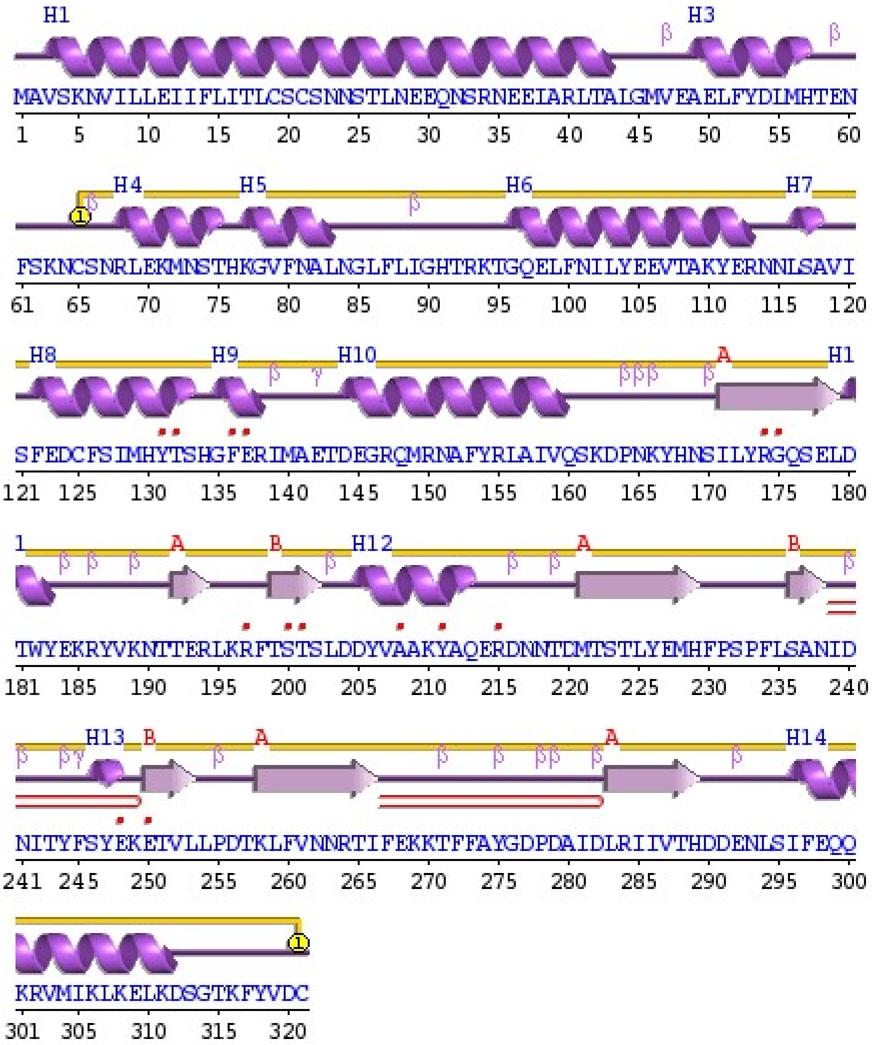

Figure S4B. LIGPLOT *Lb_340*

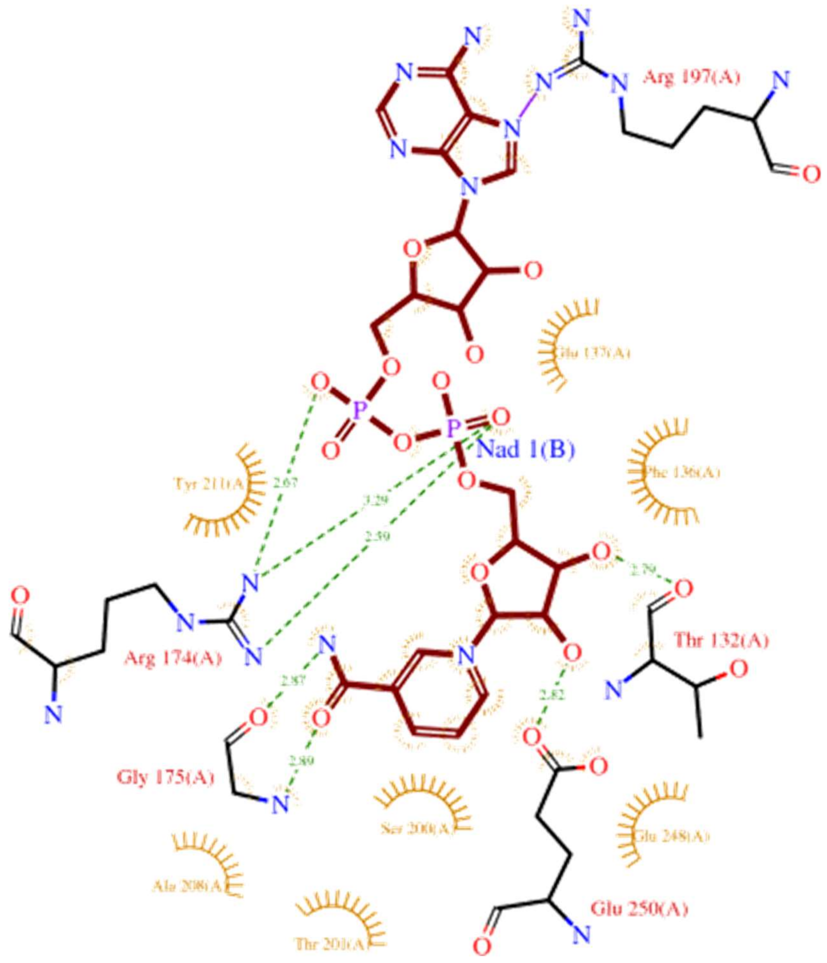

Figure S5A. SS Plot *Lh_005*

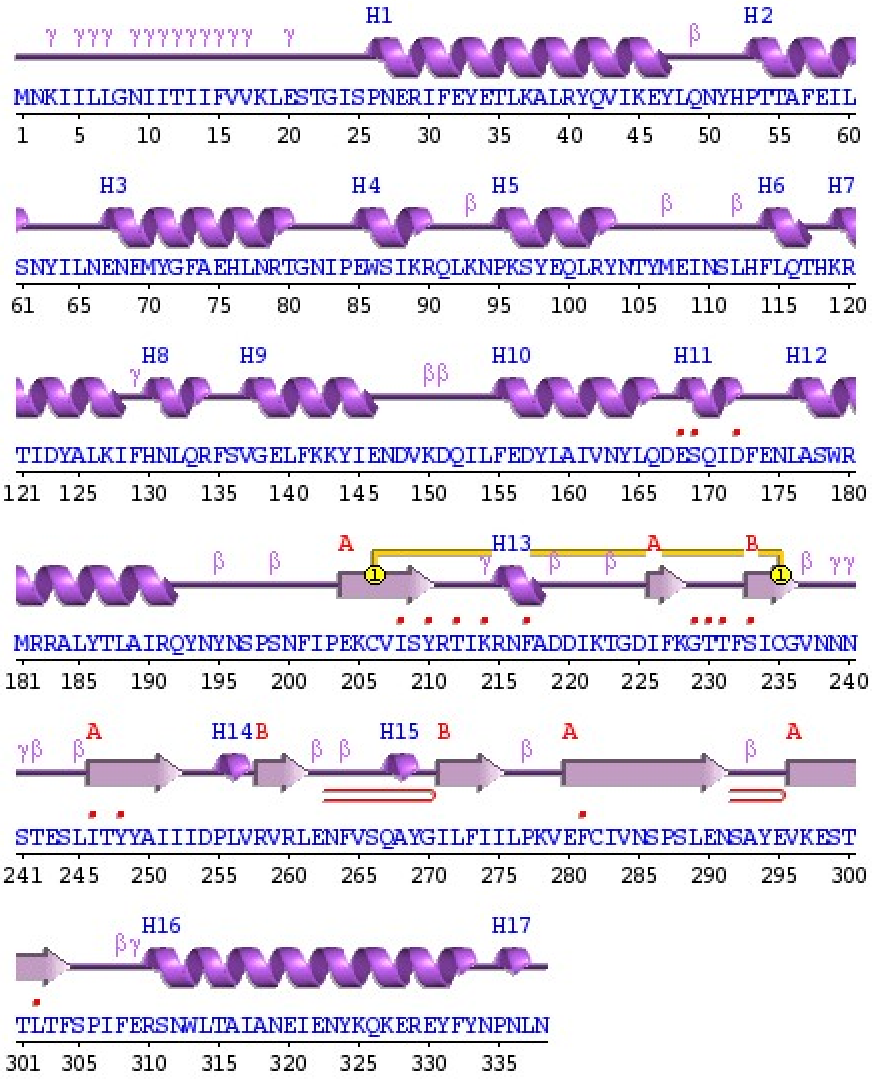

Figure S5B. LIGPLOT *Lh_005*

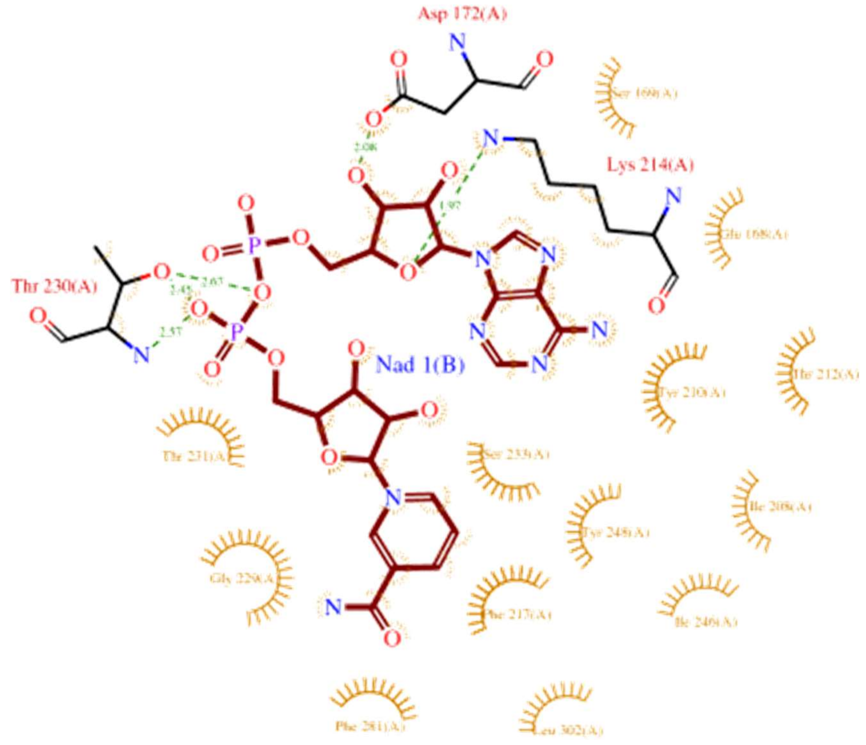

Table S1. Conserved domains in proteins found in the *L. boulardi* and *L. heterotoma* venom particles.
Conserved domains identified in *L. boulardi* (left) and *L. heterotoma* (right) and the corresponding number of proteins found with the domains are shown. A total of 358 and 290 conserved domains were found in *L. boulardi* and *L. heterotoma* proteome, respectively. There are 110 domains common to both species. Domains common to both species are in bold. Domains found only in *L. boulardi* are in blue while domains found only in *L. heterotoma* are in red.

CDD Hit (Lb) - Concise (total NR CD: 358)		
Accession	Protein Count	Short Name
**cl02570**	6	**RhoGAP superfamily**
cl38936	4	P-loop_NTPase superfamily
cl33700	4	PHA02927 superfamily
**cl37647**	4	**Myosin_tail_1 superfamily**
cl22853	3	Motor_domain superfamily
cl11960	3	Ig superfamily
**cd00063**	3	**FN3**
**cl00445**	3	**Iso_dh superfamily**
**pfam00191**	3	**Annexin**
**pfam01105**	3	**EMP24_GP25L**
**cl39281**	3	**ADAMTS_CR_2 superfamily**
cl00281	2	metallo-dependent_hydrolases superfamily
cl13995	2	MPP_superfamily superfamily
**cl31217**	2	**fa_ox_alpha_mit superfamily**
COG2303	2	BetA
cl00388	2	Thioredoxin_like superfamily
**cd00051**	2	**EFh**
**cl36495**	2	**PTZ00009 superfamily**
cl37091	2	DnaJ_bact superfamily
cl00226	2	nuc_hydro superfamily
**cl36828**	2	**ER_PDI_fam superfamily**
cl02574	2	Annexin superfamily
cd00033	2	CCP
**cl17169**	2	**RRM_SF superfamily**
**cd00751**	2	**thiolase**
cd06526	2	metazoan_ACD
**cl28691**	2	**PLN02872 superfamily**
cd19601	2	serpin42Da-like
**cl02828**	2	**Calreticulin superfamily**
pfam00080	2	Sod_Cu

CDD Hit (Lh) - Concise (total NR CD: 290)		
Accession	Protein Count	Short Name
**cl14813**	14	**GluZincin superfamily**
**cl02570**	5	**RhoGAP superfamily**
**cd00063**	4	**FN3**
cd00882	4	Ras_like_GTPase
**cl33172**	4	**PTZ00184 superfamily**
cd08662	4	M13
cd11328	3	AmyAc_maltase
pfam00173	3	Cyt-b5
cl34640	3	YeeP superfamily
**cl00445**	2	**Iso_dh superfamily**
cl44230	2	COG5412 superfamily
pfam00261	2	Tropomyosin
**cl36828**	2	**ER_PDI_fam superfamily**
**pfam00153**	2	**Mito_carr**
**cd20628**	2	**CYP4**
**pfam02036**	2	**SCP2**
cd03401	2	SPFH_prohibitin
**COG0612**	2	**PqqL**
**cl36495**	2	**PTZ00009 superfamily**
**cd00051**	2	**EFh**
**cl37647**	2	**Myosin_tail_1 superfamily**
COG1100	2	Gem1
**cl21454**	2	**NADB_Rossmann superfamily**
**cd10224**	2	**ASKHA_NBD_actin**
**cl17169**	2	**RRM_SF superfamily**
**cl28691**	2	**PLN02872 superfamily**
pfam03820	2	SFXNs
cl38192	2	Peptidase_M13_N superfamily
**cl16144**	2	**Diedel superfamily**
cl45980	2	RhoGAP superfamily

cl31127	2	PABP-1234 superfamily
**cl00064**	**2**	**ZnMc superfamily**
cl02098	2	14-3-3 superfamily
cl01033	1	Ribosomal_L35Ae superfamily
**cd02983**	**1**	**P5_C**
**cd03001**	**1**	**PDI_a_P5**
cl37779	1	A_deaminase_N superfamily
pfam07749	1	ERp29
**cl17346**	**1**	**Trehalase superfamily**
pfam00755	1	Carn_acyltransf
pfam16484	1	CPT_N
**COG0612**	**1**	**PqqL**
**pfam01158**	**1**	**Ribosomal_L36e**
cd01417	1	Ribosomal_L19e_E
cd04030	1	C2C_KIAA1228
cd04050	1	C2B_Synaptotagmin-like
cl14603	1	C2 superfamily
PTZ00189	1	PTZ00189
**cl44316**	**1**	**PTZ00027 superfamily**
**cd00176**	**1**	**SPEC**
**pfam08726**	**1**	**EFhand_Ca_insen**
smart00150	1	SPEC
**pfam03896**	**1**	**TRAP_alpha**
COG0457	1	TPR
pfam02990	1	EMP70
**cl00314**	**1**	**rpsJ superfamily**
cl46412	1	Lamp superfamily
**pfam00153**	**1**	**Mito_carr**
smart00299	1	CLH
pfam00637	1	Clathrin
pfam13838	1	Clathrin_H_link
pfam09268	1	Clathrin-link
pfam01394	1	Clathrin_propel
pfam13499	1	EF-hand_7
PTZ00219	1	PTZ00219
cd14103	1	STKc_MLCK
cl31754	1	PTZ00121 superfamily

cl45768	2	gly_rich_SclB superfamily
cl21522	1	FN3 superfamily
**cd19941**	**1**	**TIL**
pfam01826	1	TIL
**pfam00041**	**1**	**fn3**
**PRK09281**	**1**	**PRK09281**
cl02796	1	Cyt_c_Oxidase_VIIa superfamily
**cd13156**	**1**	**KOW_RPL6**
**pfam03868**	**1**	**Ribosomal_L6e_N**
cd06592	1	GH31_NET37
**PTZ00106**	**1**	**PTZ00106**
**cl44413**	**1**	**Vitellogenin_N superfamily**
**pfam09172**	**1**	**Vit_open_b-sht**
**cl47031**	**1**	**VWD superfamily**
**cl05778**	**1**	**DUF1081 superfamily**
cl27196	1	ATG17_like superfamily
**cl36873**	**1**	**aconitase_mito superfamily**
TIGR01106	1	ATPase-IIC_X-K
**cd00922**	**1**	**Cyt_c_Oxidase_IV**
smart00060	1	FN3
**pfam05817**	**1**	**Ribophorin_II**
**cd02083**	**1**	**P-type_ATPase_SERCA**
**cl42391**	**1**	**SNU13 superfamily**
**pfam00274**	**1**	**Glycolytic**
**pfam01015**	**1**	**Ribosomal_S3Ae**
**COG0055**	**1**	**AtpD**
PTZ00072	1	PTZ00072
PRK08256	1	PRK08256
cd00926	1	Cyt_c_Oxidase_VIb
**cd01869**	**1**	**Rab1_Ypt1**
cd03344	1	GroEL
PTZ00187	1	PTZ00187
pfam07766	1	LETM1_RBD
cl00307	1	Thiamine_BP superfamily
cl00017	1	Cyt_c_Oxidase_VIa superfamily
**cd01337**	**1**	**MDH_glyoxysomal_mitochondrial**
**cl31217**	**1**	**fa_ox_alpha_mit superfamily**

cl31759	1	PTZ00341 superfamily
**cd05371**	**1**	**HSD10-like_SDR_c**
cd14867	1	uS7_Eukaryote
COG3386	1	YvrE
cl45446	1	SSL_N superfamily
**pfam00213**	**1**	**OSCP**
**PTZ00069**	**1**	**PTZ00069**
**pfam05817**	**1**	**Ribophorin_II**
cl47026	1	LRRCT superfamily
cl49619	1	beta-trefoil_MIR superfamily
cd01158	1	SCAD_SBCAD
**PRK09281**	**1**	**PRK09281**
**PTZ00335**	**1**	**PTZ00335**
cd05236	1	FAR-N_SDR_e
pfam03015	1	Sterile
cd06105	1	ScCit1-2_like
cl36499	1	PTZ00037 superfamily
cl02823	1	phosphagen_kinases superfamily
PRK05899	1	PRK05899
cd07141	1	ALDH_F1AB_F2_RALDH1
cl31511	1	PHA02831 superfamily
cd08156	1	catalase_clade_3
**PLN00036**	**1**	**PLN00036**
cl17171	1	PH-like superfamily
pfam07850	1	Renin_r
cl28910	1	MFS superfamily
cl47492	1	Serpin superfamily
cd14958	1	NHL_PAL_like
cl00330	1	Ribosomal_S8 superfamily
COG3250	1	LacZ
pfam17753	1	Ig_mannosidase
pfam17786	1	Mannosidase_ig
cl31020	1	sbcc superfamily
**pfam04597**	**1**	**Ribophorin_I**
**cl00322**	**1**	**Ribosomal_L1 superfamily**
COG1132	1	MdlB
**cl33172**	**1**	**PTZ00184 superfamily**

**pfam01198**	**1**	**Ribosomal_L31e**
**cd05353**	**1**	**hydroxyacyl-CoA-like_DH_SDR_c-like**
**cl28571**	**1**	**PLN02864 superfamily**
cl01629	1	TPP_enzymes superfamily
**pfam00635**	**1**	**Motile_Sperm**
**PTZ00129**	**1**	**PTZ00129**
cd12151	1	F1-ATPase_gamma
**cl29613**	**1**	**PTZ00103 superfamily**
**cd07306**	**1**	**Porin3_VDAC**
cl16912	1	MDR superfamily
**pfam00183**	**1**	**HSP90**
cl47785	1	COX6C superfamily
pfam10206	1	WRW
**cd00751**	**1**	**thiolase**
**cl36523**	**1**	**PTZ00169 superfamily**
**pfam00992**	**1**	**Troponin**
**TIGR01973**	**1**	**NuoG**
**cl08544**	**1**	**NADH_dhqG_C superfamily**
cd22209	1	EMC10
cd17358	1	MFS_GLUT6_8_Class3_like
cl17190	1	NK superfamily
cl36077	1	PRK11892 superfamily
pfam12037	1	ATAD3_N
cd19512	1	RecA-like_ATAD3-like
cd01865	1	Rab3
cl40741	1	DAO superfamily
PTZ00139	1	PTZ00139
cd01378	1	MYSc_Myo1
pfam06017	1	Myosin_TH1
smart00015	1	IQ
**pfam04597**	**1**	**Ribophorin_I**
**cd01161**	**1**	**VLCAD**
cl02542	1	DnaJ superfamily
**pfam13927**	**1**	**Ig_3**
**smart00410**	**1**	**IG_like**
cl05735	1	DUF1075 superfamily
pfam05187	1	ETF_QO

pfam03712	1	Cu2_monoox_C
pfam01082	1	Cu2_monooxygen
**PTZ00178**	1	**PTZ00178**
cd02992	1	PDI_a_QSOX
pfam04777	1	Evr1_Alr
cl39711	1	FAD_SOX superfamily
cl39497	1	QSOX_Trx1 superfamily
cl34873	1	COG5028 superfamily
**PTZ00068**	1	**PTZ00068**
cd00170	1	SEC14
**pfam01198**	1	**Ribosomal_L31e**
**pfam00183**	1	**HSP90**
cd16927	1	HATPase_Hsp90-like
cl36852	1	CDC48 superfamily
**cd00922**	1	**Cyt_c_Oxidase_IV**
cd13314	1	PH_Rpn13
**pfam00992**	1	**Troponin**
PTZ00160	1	PTZ00160
cl02130	1	Got1 superfamily
pfam06441	1	EHN
cl21494	1	Abhydrolase superfamily
cd06562	1	GH20_HexA_HexB-like
pfam14845	1	Glycohydro_20b2
**cd13156**	1	**KOW_RPL6**
**pfam03868**	1	**Ribosomal_L6e_N**
pfam03345	1	DDOST_48kD
cl41025	1	Intu_longin_1 superfamily
cd22912	1	HFD_H4
cd06768	1	PDZ_NHERF-like
cd07132	1	ALDH_F3AB
pfam06664	1	MIG-14_Wnt-bd
cl02720	1	PB1 superfamily
cd02340	1	ZZ_NBR1_like
cd14320	1	UBA_SQSTM
cl35718	1	PRK08581 superfamily
**cd20628**	1	**CYP4**
pfam07970	1	COPIIcoated_ERV

COG0644	1	FixC
PLN03072	1	PLN03072
cl29687	1	PLN00035 superfamily
pfam00287	1	Na_K-ATPase
**PTZ00096**	1	**PTZ00096**
PRK06074	1	PRK06074
**pfam00213**	1	**OSCP**
**PLN00210**	1	**PLN00210**
cl00075	1	HATPase superfamily
cl31523	1	PHA02954 superfamily
**pfam04758**	1	**Ribosomal_S30**
**cl28922**	1	**Ubl1_cv_Nsp3_N-like superfamily**
**PTZ00178**	1	**PTZ00178**
**cd21207**	1	**CH_dMP20-like**
**pfam00402**	1	**Calponin**
cd08300	1	alcohol_DH_class_III
cl35830	1	asnB superfamily
pfam01776	1	Ribosomal_L22e
cl00175	1	alpha-crystallin-Hsps_p23-like superfamily
PLN00129	1	PLN00129
pfam10276	1	zf-CHCC
cd09404	1	LIM1_MLP84B_like
pfam15936	1	DUF4749
cd00923	1	Cyt_c_Oxidase_Va
COG0563	1	Adk
cl10760	1	NDUF_B6 superfamily
pfam00313	1	CSD
cd03505	1	Delta9-FADS-like
**pfam01395**	1	**PBP_GOBP**
**PTZ00335**	1	**PTZ00335**
cl47968	1	ApoLp-III superfamily
pfam01246	1	Ribosomal_L24e
cl10848	1	TMEM214 superfamily
PTZ00083	1	PTZ00083
**pfam01105**	1	**EMP24_GP25L**
cl30550	1	PLN00154 superfamily
pfam05529	1	Bap31

pfam13850	1	ERGIC_N
cl45890	1	GT_LH superfamily
PTZ00067	1	PTZ00067
pfam06585	1	JHBP
**COG0055**	1	**AtpD**
PLN00035	1	PLN00035
cd11056	1	CYP6-like
cl38199	1	EzrA superfamily
pfam05404	1	TRAP-delta
cl23718	1	ALP_like superfamily
COG0605	1	SodA
**cd14824**	1	**Longin**
**cd15866**	1	**R-SNARE_SEC22**
**cd13194**	1	**FERM_C_ERM**
**smart00295**	1	**B41**
**pfam00769**	1	**ERM_C**
**pfam20492**	1	**ERM_helical**
cl27313	1	DUF229 superfamily
COG0045	1	SucC
PTZ00084	1	PTZ00084
pfam01496	1	V_ATPase_I
pfam00736	1	EF1_GNE
cd10308	1	GST_C_eEF1b_like
pfam06645	1	SPC12
cd07306	1	**Porin3_VDAC**
COG1131	1	CcmA
cd18091	1	SpoU-like_TRM3-like
cd05833	1	Ribosomal_P2
smart00702	1	P4Hc
PTZ00191	1	PTZ00191
cd13761	1	TGF_beta_BMP5_like
cl46657	1	TGFb_propeptide superfamily
**PTZ00106**	1	**PTZ00106**
cl21590	1	PMT_2 superfamily
**pfam04758**	1	**Ribosomal_S30**
**cl28922**	1	**Ubl1_cv_Nsp3_N-like superfamily**
cl36705	1	rad4 superfamily

pfam18035	1	Bap31_Bap29_C
cl00274	1	ML superfamily
pfam05680	1	ATP-synt_E
PTZ00180	1	PTZ00180
COG1038	1	PycA
cd14909	1	MYSc_Myh1_insects_crustaceans
pfam02736	1	Myosin_N
pfam02939	1	UcrQ
pfam10660	1	MitoNEET_N
smart00704	1	ZnF_CDGSH
**PTZ00141**	1	**PTZ00141**
**cl30355**	1	**PLN02272 superfamily**
**cl00314**	1	**rpsJ superfamily**
**pfam01593**	1	**Amino_oxidase**
cd00924	1	Cyt_c_Oxidase_Vb
**cd13194**	1	**FERM_C_ERM**
**smart00295**	1	**B41**
**pfam00769**	1	**ERM_C**
**pfam20492**	1	**ERM_helical**
smart00202	1	SR
pfam00530	1	SRCR
pfam13229	1	Beta_helix
smart00034	1	CLECT
cl00049	1	CUB superfamily
pfam04387	1	PTPLA
cl05199	1	ATP-synt_F6 superfamily
pfam01247	1	Ribosomal_L35Ae
**cl44316**	1	**PTZ00027 superfamily**
pfam05676	1	NDUF_B7
pfam04774	1	HABP4_PAI-RBP1
cl24679	1	IHABP4_N superfamily
COG0629	1	Ssb
**PLN00220**	1	**PLN00220**
**PTZ00148**	1	**PTZ00148**
**pfam01158**	1	**Ribosomal_L36e**
pfam03031	1	NIF
**cl02828**	1	**Calreticulin superfamily**

cl00197	1	cyclophilin superfamily
**cl36523**	1	**PTZ00169 superfamily**
**TIGR01973**	1	**NuoG**
**cl08544**	1	**NADH_dhqG_C superfamily**
**PTZ00129**	1	**PTZ00129**
smart00611	1	SEC63
pfam00226	1	DnaJ
**cl30355**	1	**PLN02272 superfamily**
pfam20145	1	ARMET_N
pfam10208	1	ARMET_C
TIGR01040	1	V-ATPase_V1_B
cl00208	1	RNase_T2 superfamily
**cd23702**	1	**eL14**
pfam02077	1	SURF4
cd22948	1	Coatomer_WDAD_alpha
pfam06957	1	COPI_C
cd00200	1	WD40
**cd10224**	1	**ASKHA_NBD_actin**
cd06901	1	lectin_VIP36_VIPL
pfam01248	1	Ribosomal_L7Ae
**pfam00635**	1	**Motile_Sperm**
**pfam13927**	1	**Ig_3**
cd00096	1	Ig
**smart00410**	1	**IG_like**
**cl49606**	1	**GH38-57_N_LamB_YdjC_SF superfamily**
**pfam01015**	1	**Ribosomal_S3Ae**
**cl42391**	1	**SNU13 superfamily**
**pfam01593**	1	**Amino_oxidase**
cl33517	1	PLN02568 superfamily
**pfam05753**	1	**TRAP_beta**
pfam04893	1	Yip1
cl34951	1	SEC21 superfamily
pfam16381	1	Coatomer_g_Cpla
pfam03911	1	Sec61_beta
cl37648	1	Adaptin_N superfamily
pfam14806	1	Coatomer_b_Cpla
pfam07718	1	Coatamer_beta_C

pfam01257	1	2Fe-2S_thioredx
cd03016	1	PRX_1cys
PTZ00173	1	PTZ00173
**PLN00036**	1	**PLN00036**
COG0678	1	AHP1
**pfam01124**	1	**MAPEG**
cl26613	1	Mitofilin superfamily
pfam01251	1	Ribosomal_S7e
PRK06075	1	PRK06075
COG1249	1	Lpd
pfam13855	1	LRR_8
**cd05371**	1	**HSD10-like_SDR_c**
**PTZ00070**	1	**PTZ00070**
pfam03248	1	Rer1
COG0513	1	SrmB
pfam02271	1	UCR_14kD
**pfam03501**	1	**S10_plectin**
pfam05071	1	NDUFA12
PTZ00158	1	PTZ00158
pfam06212	1	GRIM-19
**cd00176**	1	**SPEC**
cd11808	1	SH3_Alpha_Spectrin
**pfam08726**	1	**EFhand_Ca_insen**
pfam00435	1	Spectrin
**cl00322**	1	**Ribosomal_L1 superfamily**
**cd05356**	1	**17beta-HSD1_like_SDR_c**
**cd23702**	1	**eL14**
pfam01929	1	Ribosomal_L14e
cd01336	1	MDH_cytoplasmic_cytosolic
cl17068	1	AFD_class_I superfamily
PTZ00156	1	PTZ00156
pfam06522	1	B12D
pfam03665	1	UPF0172
cd00288	1	Pyruvate_Kinase
cd11310	1	14-3-3_1
pfam04718	1	ATP-synt_G
**pfam00191**	1	**Annexin**

pfam04495	1	GRASP55_65
cl49608	1	PDZ_canonical superfamily
cl38111	1	Atrophin-1 superfamily
cl10465	1	Peptidase_S24_S26 superfamily
pfam03200	1	Glyco_hydro_63
cl25200	1	Glyco_hydro_63N superfamily
pfam01090	1	Ribosomal_S19e
cd14829	1	Zeta-COP
PRK00290	1	dnaK
**PTZ00096**	1	**PTZ00096**
cl14782	1	RNase_H_like superfamily
cl46693	1	HSP90 superfamily
pfam07074	1	TRAP-gamma
**pfam00274**	1	**Glycolytic**
COG0545	1	FkpA
cd03127	1	tetraspanin_LEL
**cd01337**	1	**MDH_glyoxysomal_mitochondrial**
cd11286	1	ADF_cofilin_like
PTZ00254	1	PTZ00254
cl24635	1	40S_SA_C superfamily
cd05646	1	M20_AcylaseI_like
cl14876	1	Zinc_peptidase_like superfamily
cl03452	1	DAD superfamily
**cd01161**	1	**VLCAD**
**cl36873**	1	**aconitase_mito superfamily**
PLN00191	1	PLN00191
cl09933	1	ACAD superfamily
**PTZ00141**	1	**PTZ00141**
pfam04733	1	Coatomer_E
cd01926	1	cyclophilin_ABH_like
**cd21207**	1	**CH_dMP20-like**
**pfam00402**	1	**Calponin**
pfam00827	1	Ribosomal_L15e
**PTZ00148**	1	**PTZ00148**
cd03577	1	NTR_TIMP_like
cl42388	1	PPP1R42 superfamily
cl34836	1	LRR superfamily

cl05413	1	NDUF_B8 superfamily
cl31858	1	PLN02284 superfamily
TIGR01310	1	uL30_euk
pfam05873	1	Mt_ATP-synt_D
PLN02681	1	PLN02681
**pfam03896**	1	**TRAP_alpha**
**PTZ00069**	1	**PTZ00069**
cl28957	1	chap_CCT_zeta superfamily
PTZ00054	1	PTZ00054
pfam05405	1	Mt_ATP-synt_B
pfam07978	1	NIPSNAP
cd19852	1	FABP_pancrustacea
pfam07347	1	CI-B14_5a
PRK06411	1	PRK06411
pfam02167	1	Cytochrom_C1
**PTZ00068**	1	**PTZ00068**
**PTZ00135**	1	**PTZ00135**
cd03470	1	Rieske_cytochrome_bc1
pfam02921	1	UCR_TM
pfam09165	1	Ubiq-Cytc-red_N
cl33442	1	PLN02252 superfamily
**cl46848**	1	**PTZ00428 superfamily**
pfam01294	1	Ribosomal_L13e
pfam00379	1	Chitin_bind_4
cd09913	1	EHD
cl41352	1	MSCRAMM_ClfA superfamily
pfam16880	1	EHD_N
cl20240	1	MRJP superfamily
**pfam11721**	1	**Malectin**
**cl17346**	1	**Trehalase superfamily**
cl33185	1	PTZ00443 superfamily
cl36508	1	PTZ00102 superfamily
pfam05197	1	TRIC
**cl49606**	1	**GH38-57_N_LamB_YdjC_SF superfamily**
**cd02983**	1	**P5_C**
**cd03001**	1	**PDI_a_P5**
pfam00445	1	Ribonuclease_T2

**cl46848**	1	**PTZ00428 superfamily**
PTZ00241	1	PTZ00241
cd00073	1	H15
**PTZ00070**	1	**PTZ00070**
cd22910	1	HFD_H2B
cl00470	1	AKR_SF superfamily
cl39129	1	Stt3 superfamily
**PLN00210**	**1**	**PLN00210**
TIGR01042	1	V-ATPase_V1_A
cd04457	1	S1_S28E
PTZ00155	1	PTZ00155
cd05831	1	Ribosomal_P1
**cl44413**	1	**Vitellogenin_N superfamily**
**pfam09172**	1	**Vit_open_b-sht**
cd06263	1	MAM
**cl47031**	1	**VWD superfamily**
pfam01033	1	Somatomedin_B
**cl05778**	1	**DUF1081 superfamily**
cl35229	1	PRK03918 superfamily
**cd02083**	1	**P-type_ATPase_SERCA**
cl42898	1	PTZ00180 superfamily
cl00931	1	Ribosomal_S6e superfamily
cd21159	1	XendoU
cl41472	1	ser_rich_anae_1 superfamily
cl21585	1	RPN5 superfamily
pfam07679	1	I-set
**pfam00041**	1	**fn3**
PTZ00134	1	PTZ00134
cd03015	1	PRX_Typ2cys
**cl29613**	1	**PTZ00103 superfamily**
pfam03661	1	TMEM33_Pom33
cl37675	1	5_nucleotid_C superfamily
**cl21454**	1	**NADB_Rossmann superfamily**
cl19519	1	FKBP_C superfamily
cd06902	1	lectin_ERGIC-53_ERGL
cl47032	1	BAG superfamily
cl34456	1	BetA superfamily

cl34844	1	EnvC superfamily
**cd14824**	1	**Longin**
**cd15866**	1	**R-SNARE_SEC22**
COG2025	1	FixB
PRK13596	1	PRK13596
pfam07047	1	OPA3
pfam01992	1	vATP-synt_AC39
pfam10249	1	NDUFB10
pfam02516	1	STT3
pfam01956	1	EMC3_TMCO1
cl40971	1	CagA_N superfamily
cl10759	1	NDUF_B5 superfamily
cl36517	1	PTZ00144 superfamily
pfam01813	1	ATP-synt_D
cl36779	1	3a01203 superfamily
COG0567	1	SucA
pfam04800	1	NDUS4
cl10749	1	ApoO superfamily
**cl11399**	1	**HP superfamily**
cl10013	1	Glycosyltransferase_GTB-type superfamily
cl04196	1	MPC superfamily
**pfam05753**	1	**TRAP_beta**
cl47805	1	Snf7 superfamily
**cd05380**	1	**CAP_euk**
cl10447	1	GH18_chitinase-like superfamily
cl00750	1	PRK00977 superfamily
COG0465	1	HflB
cd01868	1	Rab11_like
**cl11403**	1	**pepsin_retropepsin_like superfamily**
**cl00064**	1	**ZnMc superfamily**
**cl39281**	1	**ADAMTS_CR_2 superfamily**
cl28899	1	DEAD-like_helicase_N superfamily
**cl12117**	1	**JHBP superfamily**
cl35170	1	PRK01889 superfamily
cl24486	1	Myofilin superfamily
cl29770	1	PRK00409 superfamily
cd01056	1	Euk_Ferritin

**cl14813**	**1**	**GluZincin superfamily**
**cl11399**	**1**	**HP superfamily**
cd09601	1	M1_APN-Q_like
**cd01869**	**1**	**Rab1_Ypt1**
**cd05380**	**1**	**CAP_euk**
**PLN00220**	**1**	**PLN00220**
**cl16144**	**1**	**Diedel superfamily**
cd06603	1	GH31_GANC_GANAB_alpha
cd14752	1	GH31_N
cd01866	1	Rab2
cl37108	1	leuko_A4_hydro superfamily
cl47529	1	Peptidase_S9 superfamily
cl35903	1	PRK10263 superfamily
cl47615	1	TIMP superfamily
cl46410	1	GBP superfamily
cd01862	1	Rab7
**cl11403**	**1**	**pepsin_retropepsin_like superfamily**
cd04382	1	RhoGAP_MgcRacGAP
cd20821	1	C1_MgcRacGAP
pfam00450	1	Peptidase_S10
cl25547	1	Caldesmon superfamily
cd04151	1	Arl1
cl09927	1	S1_like superfamily
cd00879	1	Sar1
cd01860	1	Rab5_related
pfam11938	1	DUF3456
cl24152	1	Mucin_bdg superfamily
**pfam11721**	**1**	**Malectin**
**pfam01124**	**1**	**MAPEG**
cd03072	1	PDI_b'_ERp44
cd03070	1	PDI_b_ERp44
**PTZ00135**	**1**	**PTZ00135**
**pfam03501**	**1**	**S10_plectin**
cd04105	1	SR_beta
pfam04756	1	OST3_OST6
**cl12117**	**1**	**JHBP superfamily**
**cd19941**	**1**	**TIL**

cl45899	1	ClyA-like superfamily

cl32418	1	PRK09819 superfamily
cl38926	1	serpin superfamily
pfam01757	1	Acyl_transf_3
cl05112	1	Peptidase_A17 superfamily
cl40470	1	ps-ssRNAv_RdRp-like superfamily
cl40017	1	DUF5641 superfamily
cl25838	1	DUF1373 superfamily
cl05412	1	Ac81 superfamily
pfam13279	1	4HBT_2
cl25603	1	CwlO1 superfamily
pfam03134	1	TB2_DP1_HVA22
cd20433	1	Tudor_TDRD11
smart00318	1	SNc
cl00140	1	SNc superfamily
cl09558	1	TPD52 superfamily
**cd05356**	1	**17beta-HSD1_like_SDR_c**
COG4221	1	YdfG
pfam05154	1	TM2
**pfam01395**	1	**PBP_GOBP**
cl20391	1	DUF2181 superfamily
cd13529	1	PBP2_transferrin
cl21456	1	Periplasmic_Binding_Protein_Type_2 superfamily
PRK08278	1	PRK08278
COG3255	1	SCP2
pfam03208	1	PRA1
cl48181	1	DDE_Tnp_4 superfamily
**cd05353**	1	**hydroxyacyl-CoA-like_DH_SDR_c-like**
**cl28571**	1	**PLN02864 superfamily**
**pfam02036**	1	**SCP2**
cl00054	1	DSRM_SF superfamily
cl36533	1	PTZ00248 superfamily
cd10230	1	ASKHA_NBD_HSP70_HYOU1

Table S2. *Leptopilina boulardi* proteins with high confidence homology to toxins.

L. boulardi (*Lb*) proteins that identified one or more toxins in the BLAST search are shown within their toxin category. The percent query coverage ranges between 44% and 99%, except for g3260.t1, where the query coverage is 16%. The predicted conserved domains in the query sequence are indicated in the *Lb* CDD column. *Lb* AA refers to the number of amino acids in the *Lb* query protein. Cys refers to number of cysteine residues. SP stands for signal peptide and TM for transmembrane domain. Their absence (N = no), or presence (Y= yes) are indicated. The UniProt accession ID, the E-value of the top hit, and the CDD ID of the toxin protein identified are also shown (purple). Proteins where the CDD IDs between the query and subject sequences matched are color-coded and bolded.

Toxin Category	*Lb* protein	*Lb* CDD	*Lb* AA	Cys	SP	TM	Toxin hit		Toxin CDD
	Query						Accession ID	BLAST E-value	
Serpin-like	g107.t1	**cl38926** serpin (0)	402	2	Y	N	A0A0K8RCY5	1.18E-45	**cl38926** (0)
	g21089.t1	**cl38926** serpin (5.74E-122)	391	2	N	N	A0A0K8RCY5	3.25E-37	**cl38926** (0)
	g26546.t1	**cl38926** serpin (1.16E-14)	92	1	N	N	A0A0K8RCY5	3.14E-08	**cl38926** (0)
	g11786.t1	**cl38926** serpin (2.23E-74)	911	22	N	Y	A0A0K8RCY5	4.73E-18	**cl38926** (0)
	g1397.t1	**cl20226** trypsin inhibitor-like cysteine rich domain (1.83E-03 – 2.31E-03)	146	15	N	N	Q8T0W0	7.06E-07	**cl20226** (2.84E-10)
MEP/MPP/ disintegrin	g25658.t1	**cl14813** Gluzincin peptidase family (5.38E-18)	89	3	N	N	W4VS99	2.87E-09	**cl14813** (0)
	g23282.t1	**cl00064** zinc-dependent metalloprotease (7.83E-20)	313	4	Y	N	B5AJT4	1.67E-16	**cl00064** (4.59E-32)
	g8353.t1	**cl00064** zinc-dependent metalloprotease (9.50E-33)	242	9	N	N	B5AJT4<br>Q698K8	1.13E-08<br>1.72E-07	**cl00064** (4.59E-32)<br>**cl00064** (1.62E-99)
CRiSP	g882.t1	**cl00133** CAP (cysteine-rich secretory proteins, antigen 5, and pathogenesis-related 1 proteins) domain (3.42E-19)	160	5	N	N	Q91055	2.72E-06	**cl00133** (1.12E-71)
Ca^{2+} binding	g699.t1	cl34916 Ca^{2+} binding protein EF-hand superfamily (1.35E-20)	210	0	N	N	Q8AY75	1.28E-07	cl33172 calmodulin (3.61E-39)
Lipase	g13378.t1	**cl28691** triacylglycerol lipase (7.76E-49)	352	4	N	N	J3SDX8	3.53E-54	**cl28691** (5.59E-54)
	g20934.t1	**cl28691** triacylglycerol lipase (8.18E-41)	345	2	N	N	J3SDX8	1.96E-43	**cl28691** (5.59E-54)
Trehalase	g6244.t1	**cl17346** trehalase (2.78E-176)	505	4	N	N	Q8MMG9	0	**cl17346** (0)
Aspartic peptidase	g2172.t1	**cl11403** cellular and retroviral pepsin-like aspartate proteases (0)	364	6	N	N	Q18DC8<br>Q18DC9	8.08E-110<br>2.16E-98	**cl11403** (0)
Serine protease (*Lb* only)	g3398.t1	**cl11960** Ig (4.10E-08 – 7.64E-23)	1047	13	N	N	Q1HLC0	1.24E-54	**cl11960** (3.01E-36 – 2.78E-52)
	g2472.t1	**cl21456** type 2 periplasmic binding fold (1.50E-24, 2.98E-52)	708	29	Y	N	K9IMD0	1.26E-25	**cl21456** (0)
5'-nucleotidase (*Lb* only)	g24769.t1	**cl13995** metallophosphatase (9.38E-106)	251	2	N	N	F8S0Z7	1.34E-58	**cl13995** (1.29E-151) with cl37675 5'-nucleotidase (2.31E-46)
	g6755.t1	**cl13995** metallophosphatase (7.79E-46)	121	3	Y	N	F8S0Z7	6.46E-26	**cl13995** (1.29E-151) with cl37675 5'-nucleotidase (2.31E-46)
	g9134.t1	**cl37675** 5'-nucleotidase C-terminal (1.78E-10)	65	2	N	N	F8S0Z7	6.53E-12	cl13995 (1.29E-151) with **cl37675** (2.31E-46)
Other enzymes (*Lb* only)	g4648.t1	**cl11399** histidine phosphatase (6.42E-30)	355	1	N	N	Q5BLY5	1.48E-80	**cl11399** (2.48E-24)
	g3260.t1	**cl00388** thioredoxin-like (4.05E-101)	242	3	N	N	P0CV91	8.21E-10	**cl00388** (5.88E-17)
	g14621.t1	cl08270 serine carboxypeptidase (1.44E-128)	752	3	N	N	C9WMM5	0	cl47550 serine carboxypeptidase (2.53E-134)
	g1968.t1	cl21494 alpha/beta hydrolase (4.15E-48)	166	2	N	N	B2D0J4	4.71E-58	cl47529 prolyl oligopeptidase (3.71E-52)

Table S3. *Leptopilina heterotoma* proteins with high confidence homology to toxins.

L. heterotoma (*Lh*) proteins that identified one or more toxins in the BLAST search are shown within their toxin category. The percent query coverage for non-MEP toxins ranged between 65%-100%, except GAJC01009588.1_5, which showed query coverage of 28%. For the toxins in the MEP category, percent query coverage ranged between 10% - 98%. The predicted conserved domains in the query sequence are indicated in the *Lh* CDD column. Proteins where the CDD IDs between the query and subject sequences matched are shaded in blue. *Lh* AA refers to the number of amino acids in the *Lh* query protein. Cys refers to number of cysteine residues. SP stands for signal peptide and TM for transmembrane domain. Their absence (N = no), or presence (Y= yes) are indicated. The UniProt accession ID, the E-value of the top hit, and the CDD ID of the toxin protein identified are also shown (purple header).

Toxin Category	*Lh* protein	*Lh* CDD	*Lh* AA	Cys	SP	TM	Toxin hit		Toxin CDD
	Query						Accession ID	BLAST E-value	
Serpin-like	GAJC01002499.1_10	**cl20226** trypsin inhibitor-like cysteine rich domain (1.56E-05 – 1.56E-06)	168	21	Y	N	P0DM55	3.70E-07	**cl20226** (1.32E-08 – 3.62E-12)
MEP/MPP/ disintegrin	GAJC01000282.1_4	**cl14813** Gluzincin peptidase family (1.72E-31)	113	3	N	N	W4VS99	2.27E-12	**cl14813** (0)
	GAJC01000870.1_33	**cl14813** Gluzincin peptidase family (1.55E-32)	463	5	N	Y	W4VS99	4.21E-07	**cl14813** (0)
	GAJC01001743.1_44	**cl14813** Gluzincin peptidase family (3.12E-81)	656	13	Y	N	W4VS99	5.49E-37	**cl14813** (0)
	GAJC01003564.1_5	**cl14813** Gluzincin peptidase family (1.04E-65)	507	9	N	N	W4VS99	1.04E-25	**cl14813** (0)
	GAJC01013609.1_18	**cl14813** Gluzincin peptidase family (2.96E-27)	642	11	Y	N	W4VS99	3.71E-09	**cl14813** (0)
	GAJC01013610.1_18	**cl14813** Gluzincin peptidase family (1.62E-28)	625	11	Y	N	W4VS99	4.73E-09	**cl14813** (0)
	GAJC01016782.1_12	**cl14813** Gluzincin peptidase family (1.71E-48)	565	7	Y	N	W4VS99	2.96E-11	**cl14813** (0)
	GAJC01017489.1_17	**cl14813** Gluzincin peptidase family (3.56E-43)	655	9	N	N	W4VS99	2.06E-10	**cl14813** (0)
	GAJC01020718.1_27	**cl14813** Gluzincin peptidase family (1.50E-26)	278	7	Y	N	W4VS99	8.58E-17	**cl14813** (0)
	GAJC01020719.1_22	**cl14813** Gluzincin peptidase family (2.78E-18)	335	6	Y	N	W4VS99	2.24E-11	**cl14813** (0)
	GAJC01020720.1_15	**cl14813** Gluzincin peptidase family (5.97E-52)	239	6	N	N	W4VS99	7.10E-24	**cl14813** (0)
	GAJC01020722.1_12	**cl14813** Gluzincin peptidase family (8.59E-41)	171	2	N	N	W4VS99	2.26E-12	**cl14813** (0)
	GAJC01020902.1_47	**cl14813** Gluzincin peptidase family (2.05E-77)	666	10	N	N	W4VS99	5.65E-24	**cl14813** (0)
	GAJC01024282.1_10	**cl14813** Gluzincin peptidase family (2.28E-21)	91	3	N	N	W4VS99	1.16E-10	**cl14813** (0)
	GAJC01028412.1_11	**cl14813** Gluzincin peptidase family (6.78E-86)	663	11	N	N	W4VS99	2.56E-29	**cl14813** (0)
	GAJC01028413.1_13	**cl14813** Gluzincin peptidase family (2.05E-80)	663	11	N	N	W4VS99	2.96E-22	**cl14813** (0)
	GAJC01029468.1_54	**cl00064** Zinc-dependent metalloprotease (1.93E-31)	904	35	Y	N	B5AJT4<br>Q698K8	2.24E-13<br>4.21E-15	**cl00064** (4.59E-32)<br>**cl00064** (1.62E-99)
	GAJC01020721.1_11	cl38192 peptidase family M13 (8.52E-15)	164	3	N	Y	W4VS99	5.20E-11	cl14813 (0)
CRiSP	GAJC01009318.1_8	**cl00133** CAP (cysteine-rich secretory proteins, antigen 5, and pathogenesis-related 1 proteins) domain family (1.59E-40)	208	7	N	N	Q91055	7.92E-10	**cl00133** (1.12E-71)
Ca^{2+} binding	GAJC01010984.1_6	**cl33172** calmodulin (2.20E-26)	149	1	N	N	Q8AY75	1.20E-22	**cl33172** calmodulin (3.61E-39)
	GAJC01011653.1_5	**cl33172** calmodulin (2.05E-20)	218	1	N	N	Q8AY75	1.83E-07	**cl33172** calmodulin (3.61E-39)
	GAJC01013138.1_9	cl34916 Ca^{2+} binding protein, EF hand superfamily (1.73E-20)	196	0	N	N	Q8AY75	2.56E-08	cl33172 calmodulin (3.61E-39)
Lipase	GAJC01027753.1_25	**cl28691** triacylglycerol lipase (3.35E-40)	398	3	Y	N	J3SDX8	2.27E-55	**cl28691** (5.59E-54)
	GAJC01017199.1_29	**cl28691** triacylglycerol lipase (1.83E-43)	380	5	Y	N	J3SDX8	4.00E-50	**cl28691** (5.59E-54)
Trehalase	GAJC01021538.1_51	**cl17346** trehalase (0)	596	4	Y	N	Q8MMG9	0	**cl17346** trehalase (0)
Aspartic peptidase	GAJC01012383.1_11	**cl11403** cellular and retroviral pepsin-like aspartate proteases (0)	383	6	Y	N	Q18DC8<br>Q18DC9	2.20E-113<br>7.67E-102	**cl11403** (0)
LAO (*Lh* only)	GAJC01010465.1_24	cl38049 flavin containing amine oxidoreductase (4.90E-46)	448	3	Y	N	O93364	3.40E-08	cl34196 monoamine oxidase (1.72E-121)
Cold shock-like (*Lh* only)	GAJC01009588.1_5	**cl09927** S1_like (5.44E-28)	250	0	N	N	P0A968	1.44E-09	**cl09927** S1_like (1.94E-52)
Icarapin-like (*Lh* only)	GAJC01009987.1_9	no CDD	206	1	Y	N	Q5BLY4<br>Q5EF78	3.00E-37	no CDD

Supplementary Table S4. Bacterial ADP-ribosyltransferase-like proteins from wasp venom particles.

Table showing details of the bacterial ADP-ribosyltransferase-like proteins from extracellular vesicle-like structures in parasitoid wasp venom. The wasp species in which the protein was identified, inhouse ID, its RefSeq accession number and the corresponding protein or transcript IDs are shown. The NCBI XP sequences were used to analyze secondary structures and NAD^+ ligand docking (Figures S2-S5). A predicted template modeling (pTM) score above 0.5 means the overall predicted fold for the complex might be similar to the true structure. The interface predicted template modeling score (ipTM) in Alphafold measures the accuracy of the predicted relative positions of the subunits within the complex. Values higher than 0.8 represent confident high-quality predictions.

Organism	Inhouse ID	RefSeq accession	Transcript/protein ID	Amino acids	Alphafold model pTM score	ipTM confidence score for NAD^+ docking
L. boulardi	*Lb_284*	XP_051165226.1	g15068.t1	487	0.6	0.91
L. boulardi	*Lb_316*	XP_051176700.1	g22788.t1	308	0.85	0.92
L. boulardi	*Lb_340*	XP_051161330.1	g26022.t1	321	0.86	0.92
L. heterotoma	*Lh_005*	XP_043484983.1	GAJC01002819.1_26	338	0.84	0.82
